# Effectiveness of iron polymaltose complex in treatment and prevention of iron deficiency anemia in children: a systematic review and meta-analysis

**DOI:** 10.7717/peerj.10527

**Published:** 2021-01-13

**Authors:** Ritzzaleena Rosli Mohd Rosli, Mohd Noor Norhayati, Shaiful Bahari Ismail

**Affiliations:** Department of Family Medicine, School of Medical Sciences, Universiti Sains Malaysia, Kubang Kerian, Kelantan, Malaysia

**Keywords:** Iron deficiency anaemia, Iron polymaltose complex OR Ferrous III OR Ferric, Children OR Paedriatric, Treatment AND iron deficiency anaemia, Iron polymaltose complex AND iron deficiency anaemia, Iron deficiency anaemia AND iron polymaltose complex AND children OR paedriatric

## Abstract

**Background:**

Iron deficiency anemia (IDA) is commonly treated with iron formulations. Despite the expanding acceptance of iron polymaltose complex (IPC) among clinicians, there is sparse and contradictory evidence regarding its efficacy in the management of IDA in children. This systematic review and meta-analysis aimed to assess the effectiveness of IPC in the treatment and prevention of IDA in children.

**Methods:**

We searched the Cochrane Central Register of Controlled Trials, MEDLINE and Epistemonikos for all randomized control trials (RCTs) comparing oral IPC with standard oral iron supplementation for the treatment or prevention of IDA in children. We independently screened the titles and abstracts of identified trials before the full text of relevant trials was evaluated for eligibility. We then independently extracted data on the methods, interventions, outcomes, and risk of bias from the included trials. A random-effects model was used to estimate the risk ratios and mean differences with 95% confidence intervals.

**Results:**

Eight trials comprising 493 randomized patients were included and analyzed using three comparison groups. The comparison group of which was used to evaluate IPC and ferrous sulphate (FS) for treatment of IDA showed that IPC is less effective in increasing Hb (MD −0.81, 95% CI −1.08 to −0.53; I^2^ = 48%, *P* < 0.001; six studies, 368 participants; high certainty of evidence), ferritin (MD −21.24, 95% CI −39.26 to −3.23, random-effects; I^2^ = 65%, *P* = 0.020; 3 studies, 183 participants; moderate certainty of evidence) and MCV levels (MD −3.20, 95% CI −5.35 to −1.05; *P* = 0.003; one study, 103 participants; low certainty of evidence). There was no difference in the occurrence of side effects between IPC and FS group (MD 0.78, 95% CI 0.47 to 1.31; I^2^ = 4%, *P* = 0.35; three studies, 274 participants; high certainty of evidence).

**Conclusions:**

There was moderate to high certainty evidence that FS is superior to IPC with a clinically meaningful difference in improving the Hb and ferritin levels in the treatment of IDA in children. There was no difference in the occurrence of gastrointestinal side effects with high certainty evidence between the IPC and FS groups. The body of evidence did not allow a clear conclusion regarding the effectiveness of IPC with iron gluconate and iron bisglycinate in the prevention and treatment of IDA. The certainty of evidence was low. Adequately powered and high-quality trials with large sample sizes that assess both hematological and clinical outcomes are required.

**PROSPERO registration number: CRD42019145020:**

## Introduction

Iron deficiency anemia (IDA) is the leading cause of anemia globally, affecting almost one-third of the world’s population. Iron deficiency was accountable for 62.6% of anemia cases worldwide in 2013, and children of both sexes under 10 years of age had the highest prevalence (Kassebaum, 2016; WHO, 2017). IDA concurrently causes 59.5% total years of life lived with disability (Kassebaum, 2016). There is strong evidence that IDA has a significant correlation with delayed growth and neurodevelopment, as well as harmful effects on child cognitive function, which may be irreversible (Auerbach & Adamson, 2016; Subramaniam & Girish, 2015).

Anemia is diagnosed when the hemoglobin (Hb) concentration in blood is below the established cut-off point. The cut-off values vary according to age, sex, elevation, smoking status, and physiological conditions such as pregnancy. According to the World Health Organization (WHO), the Hb thresholds for diagnosing anemia in children are divided into preschool age (0.5–4.99 years), lower school age (5–11.99 years) and upper school age (12–14.99 years). The threshold for upper school age is further subdivided into male and female (WHO, 2011). Additional criteria have been suggested and include adjustments according to ethnicity and population group (WHO, 2017). An internationally recommended cut-off point for diagnosing anemia in infant younger than six months has yet to be established (WHO, 2011).

Another useful marker for identifying anemia is examine the red cell indices of full blood counts. Iron deficiency causes a reduction in both mean cell hemoglobin (MCH) and mean cell volume (MCV), which known as microcytic hypochromic anemia. It has relatively good sensitivity but only occurs in longstanding IDA (Lopez et al., 2016; WHO, 2017). These results may reinforce the preliminary evidence of iron storage (Thomas et al., 2013). Nonetheless, microcytosis also presents in hemoglobinopathies, sideroblastic anemia, and may overlap with thalassemia or sickle cell disease.

Establishing iron deficiency as the cause of anemia can be done using several laboratory markers. If acute inflammation and infection are not present, serum ferritin has the highest specificity (98%) and correlates proportionately to total body iron storage (Lopez et al., 2016). The universally accepted values for diagnosing iron deficiency are below 15 µg/L for people older than five years of age and below 12 µg/L for children younger than five years (WHO, 2017). However, serum ferritin has limited diagnostic value for patients with acute or chronic inflammatory disorders, malignancy, or liver pathology. In such conditions, calculating the transferrin saturation is more accurate, and this can be done by assessing the iron level and total iron-binding capacity (Lopez et al., 2016).

In view of the various causes of IDA, it is crucial for clinicians to differentiate between isolated iron deficiency and iron deficiency with chronic disease, such as malaria, cancer, tuberculosis, and HIV (Cook, 2005). Isolated or uncomplicated IDA occurs in otherwise well individuals, commonly due to a high iron metabolisms demand, for example, during growth spurts in pediatric populations and due to the increased iron requirement by the fetus in pregnant women. Conversely, iron deficiency with chronic disease occurs due to an impairment in the supply of iron to the plasma due to inflammatory reactions from the underlying chronic diseases (McLean et al., 2009).

Iron is an indispensable trace element that the human body has primarily been designed to conserve. It predominantly presents as a heme constituent and plays an essential role in oxygen circulation, mitochondrial function, and production of energy (Subramaniam & Girish, 2015). Nearly all body iron is recycled through erythropoiesis, and the amount of iron absorbed via duodenal enterocytes is minimal (Camaschella, 2017). Hepcidin is a peptide hormone produced by the liver that plays a crucial role in ensuring systemic iron equilibrium. Hepcidin expression increases in response to increased circulating and tissue iron, inflammatory cytokines, and metabolic needs. Hepcidin transcription is suppressed by erythropoiesis expansion, iron depletion, and low oxygenation (Camaschella, 2017; Subramaniam & Girish, 2015).

The rate of intestinal iron absorption is therefore influenced by body iron stores, the erythropoietic rate, and the bioavailability of dietary iron, which explains the important role of iron supplementation in managing IDA. The recommended dose of iron supplementation is 1–2 mg/kg/day of elemental iron for the prevention of IDA and 3–6 mg/kg/day of elemental iron to treat IDA (Ozdemir, 2015; WHO, 2016). An effective treatment should increase the Hb level by at least 1 g/L after one month (Subramaniam & Girish, 2015). The effectiveness of an intervention is best monitored by assessing the patient’s Hb and ferritin levels every two to three months until normalized, and iron supplementation should be continued for at least three months (Subramaniam & Girish, 2015; WHO, 2007).

Since the 19th century, the use of oral iron formulations, especially ferrous sulphate (FS), has been reported to cure IDA (Auerbach & Adamson, 2016), and it has become the current standard treatment for this condition (Camaschella, 2017). However, more than half of patients appear to respond inadequately to oral iron therapy because of adherence issues resulting from the side effects (DeLoughery, 2019). Gastrointestinal disturbances occur due to the oxidation reduction of free intestinal irons. The non-absorbed iron is potentially toxic to the gastrointestinal mucosa due to its oxidative properties (Tolkien et al., 2015).

A meta-analysis in 2015 showed that FS increased gastrointestinal disturbances with an odds ratio of 2.32 (Tolkien et al., 2015). The most frequently reported gastrointestinal disturbances include nausea, vomiting, colicky abdominal pain, gastritis, tarry stool, and diarrhea (Cancelo-Hidalgo et al., 2013; DeLoughery, 2019; Tolkien et al., 2015). Recent studies have described a close association between oral iron and bowel inflammation, particularly in children and patients with known inflammatory bowel disease (Paganini & Zimmermann, 2017; Yilmaz & Li, 2018).

Iron polymaltose complex (IPC) was introduced to avoid these problems. It contains non-ionic polynuclear iron (III)-hydroxide cores. It is superficially surrounded by several non-covalently bound polymaltose molecules, forming a stable complex that is highly water soluble within a wide range of pH levels. However, because of its size, IPC diffuses through the mucosal membrane 40 times slower than ferrous iron (Burckhardt-Herold et al., 2007). Contrary to ferrous iron, the absorption of IPC is up to seven times better when taken with meals, and should be the recommended method of consumption (Geisser & Burckhardt, 2011; Toblli & Brignoli, 2007).

Evidence has shown that the bioavailability of ferrous iron and IPC is comparable (Jacobs, Wood & Bird, 2000). However, several contradictory studies have made the efficacy of IPC a topic of debate (Geisser, 2007). A meta-analysis in adult populations showed that there was no difference in Hb levels following treatment for IDA with either FS or IPC, but IPC was more tolerable (Toblli & Brignoli, 2007). Similar results were reported for the treatment of IDA in a pregnant population (Ortiz et al., 2011). However, homogenous evidence on IPC for treatment and prevention of IDA in pediatric populations is not well established.

The objectives of this study were therefore to determine the efficacy and safety of oral iron IPC for the prevention and treatment of IDA in infants, children, and adolescents. The aim was to provide high-quality evidence comparing oral IPC and oral ferrous iron commonly used in routine practice.

## Methods

Our systematic review was conducted according to the protocol previously published in the PROSPERO register (Registration no: CRD42019145020). The methodology and reporting were based on recommendations from the Cochrane Collaboration (Higgins et al., 2019) and the preferred reporting items for systematic reviews and meta-analyses statement (Moher et al., 2009). The evaluation was conducted according to the Grading of Recommendations Assessment, Development and Evaluation (GRADE) guidelines (Guyatt et al., 2008).

### Eligibility criteria

We considered randomized controlled trials (RCTs) comparing oral IPC with ferrous iron for the prevention or treatment of IDA. We excluded cross-over trials because the primary outcomes are considered irreversible. The participants included children of both sexes and of any ethnicity. The interventions were oral IPC supplementation in either a preventative or treatment dosage. The comparators were selected based on the availability of comparative studies conducted against IPC.

We did not impose any exclusion criteria for the trials aside from language restrictions to maximize the number of included trials. Only English language publications were included given the limitations inherent to translating non-English papers. Nonetheless, language restrictions have no influence on systematic bias in systematic review-based meta-analyses in standard medical practice (Morrison et al., 2012).

### Search strategy

We searched the Cochrane Central Register of Controlled Trials, MEDLINE, and Epistemonikos from inception to July 2019. We used the text words “iron deficiency anaemia”, “iron polymaltose complex” and “children” as well as Boolean operators like AND, OR, truncation, and wildcards for variations in words (Appendix S1). We checked the reference list of the identified RCTs and review articles to find unpublished trials or trials not identified by the electronic searches. We also searched for ongoing trials through the WHO International Clinical Trials Registry Platform and ClinicalTrials.gov.

### Selection criteria

**Figure 1:**
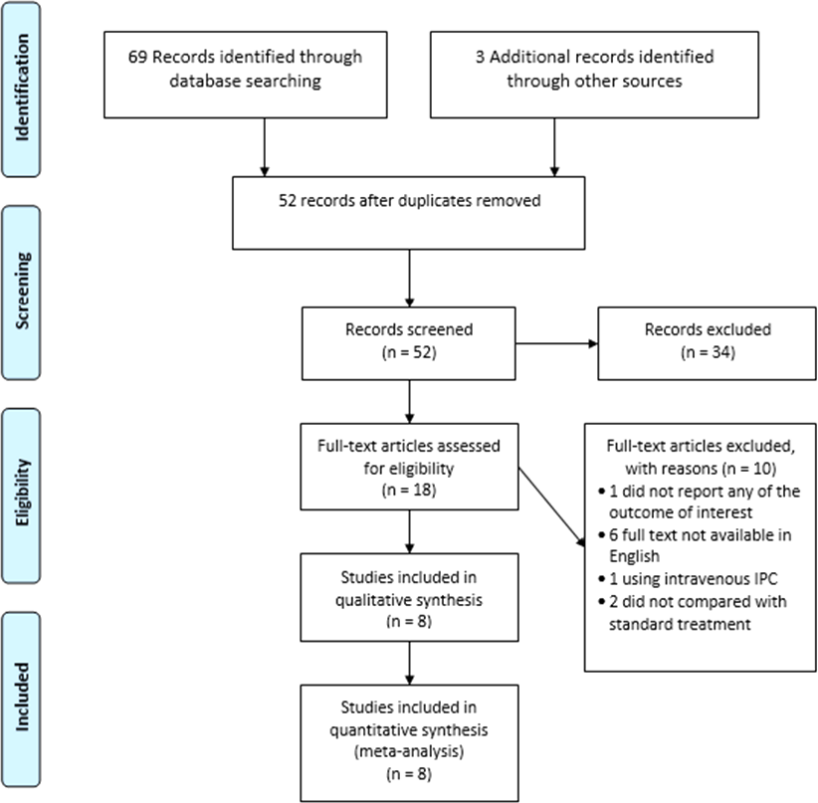
PRISMA study flow diagram.

We independently screened all the titles and abstracts identified through the implementation of the search strategy to determine the trials for further assessment. The full text of all trials that could potentially be included were screened and evaluated according to the eligibility criteria. Any reasons for exclusion were documented. Conflicts between the review authors were resolved by discussion. The study flow diagram (Fig. 1) mapped out the number of records identified, screened, included, and excluded (with reasons).

### Data extraction

Using the data extraction form, we independently extracted the key characteristics of the trials (study setting), the participants’ characteristics (age, sex, ethnicity, severity of IDA at baseline), the methodology (number of patients randomized and analyzed, duration of follow up, inclusion and exclusion criteria), the description of the intervention (preparation, dosage, duration), and the outcomes.

### Outcomes

The predefined primary outcomes included the level of Hb in g/L and serum ferritin in ng/mL or mcg/L at the end of treatment. These values were taken from the results of the full blood count and iron studies using venous blood samples that were sent to the respective laboratories and tested using the applicable local setting. The secondary outcomes were serum iron level (mcg/dL), MCV (fL), MCH (pg), and the occurrence of gastrointestinal disturbances (abdominal pain, nausea, vomiting, diarrhea and constipation) at any time during the study period after the participants had been randomized into the intervention and control groups.

### Assessment of risk of bias

All the included studies were assessed for risk of bias as outlined in the Cochrane Handbook of Systematic Reviews of Interventions (Higgins et al., 2019). We independently assessed each trial based on random sequence generation, allocation concealment, the blinding of participants and personnel, the blinding of outcome assessors, the completeness of the outcome data, the selectivity of outcome reporting, and other forms of bias. We categorized the risk of bias as low, unclear, or high.

### Statistical analysis

All the statistical analyses were carried out using Review Manager software (Review Manager, 2011). For all the included trials with continuous outcomes, we calculated the mean differences (MDs) with 95% confidence intervals (CI), and for those with dichotomous outcomes, we presented the results as a summary of the risk ratios (RR). We further reported the results using the random effects model. We pooled these measures in the meta-analyses and drew forest plots. We checked the included trials for unit of analysis errors but did not encounter any of these. For the multiple-arm studies, we only included the intervention and control groups that met the eligibility criteria in the analyses.

We assessed for obvious heterogeneity by comparing the populations, interventions, comparators, and outcomes. We then assessed the statistical heterogeneity utilizing the I^2^ statistic (Higgins et al., 2019). Thresholds for the interpretation of the I^2^ can be misleading since the importance of inconsistency depends on several factors. Heterogeneity was interpreted as follows: 0% to 40% might not be important, 30% to 60% may represent moderate heterogeneity, 50% to 90% may represent substantial heterogeneity, and 75% to 100% may indicate considerable heterogeneity (Higgins et al., 2019).

We planned to carry out subgroup analyses according to the age of the children, the duration of treatment, the dose of elemental iron, and whether it was used for the prevention or treatment of IDA. We performed a sensitivity analysis to investigate the impact of risk of bias for sequence generation and allocation concealment on the included trials.

We used the GRADE approach to assess the quality of the evidence for primary and secondary outcomes. We assessed the presence of risk of bias, inconsistent or unexplained heterogeneity, and indirectness of evidence imprecision of results (Guyatt et al., 2008). The quality of the evidence for each outcome was analyzed using GRADEpro software and sorted into four categories: high, moderate, low, and very low. These are all presented in the ‘summary of findings’ table.

## Results

### Trial selection

The electronic searches from inception until July 2019 retrieved a total of 69 records and three ongoing trials from other sources. After removing duplicate records, we screened 52 records and excluded 34 that did not meet the eligibility criteria. Of the remaining 18 trials, six were excluded due to the inaccessibility of the English full text (Amaral et al., 2012; Arvas & Gur, 2000; Borbolla et al., 2000; Haliotis & Papanastasiou, 1998; Murahovschi et al., 1987; Schmidt et al., 1985). A further four trials were excluded because one did not fulfil the eligibility criteria for the study outcomes (Sheikh, Shah & Shakir, 2017), one used intravenous IPC instead of oral IPC (Akarsu et al., 2013) and two trials did not compare IPC with a ferrous formulation (Afzal et al., 2009; Prasetyani et al., 2017). There were three records related to ongoing trials; one trial was using oral IPC (NCT, 2014) and two trials were using a different type of ferric iron and did not meet the eligibility criteria for this review (NCT, 2017; NCT, 2018). Figure 1 summarizes the results of the search strategy.

### Characteristics of the included trials

The eight trials included 493 participants. Only one trial was designed for anemia prevention in healthy infants (Jaber et al., 2010), and it was also the only trial with a population age below six months. The remaining seven trials focused on the treatment of children with IDA (Aycicek et al., 2014; Bopche et al., 2009; Kavakli et al., 2004; Name, Vasconcelos & Valzachi Rocha Maluf, 2018; Ozsurekci et al., 2015; Sozmen et al., 2003; Yasa, Agaoglu & Unuvar, 2011). Table 1 summarizes the characteristics of the included trials.

**Table 1:** Characteristic of included trials.

Studies	Location	Size (n)	Age range	Duration of treatment	Dosage (elemental iron/day)	Comparison
Jaber et al. (2010)	Community paediatric centre, Israel	105	4–6 months old healthy infants	6–8 months (until age 1)	7.5 mg/day until 6 months old, 15 mg/day from 6 months to 1 year old.	Iron gluconate
Sozmen et al. (2003)	Turkey	25	8 months–14 years old with IDA	6 months	6 mg/d in the first 3 months, then 3 mg/kg for 3 months	Ferrous sulphate
Kavakli et al. (2004)	Paediatric outpatient clinic, Ege University, Turkey.	39	6 months –15 years old with IDA	6 months	6 mg/kg/day in the first 3 months, then 2 mg/kg/day for 3 months.	Ferrous sulphate
Bopche et al. (2009)	Teaching institution with tertiary level paediatric centre, Central India	106	1-6 years old with IDA	1 month	6 mg/kg/day	Ferrous sulphate
Yasa, Agaoglu & Unuvar (2011)	Department of Paediatric Outpatient Clinic of University of Istanbul	103	7 months–17 years old with IDA	4 months	5 mg /kg/day	Ferrous sulphate
Aycicek et al. (2014)	Paediatric and Paediatric Haematology Outpatient Clinic, Harran University, Turkey	55	1–16 years old with IDA	1 month	5mg/kg/d	Ferrous sulphate
Ozsurekci et al. (2015)	Outpatient clinic in Hacettepe University Faculty of Medicine, Paediatric Haematology Unit, Turkey	40	6 months to 15 years old with IDA	2 months	6 mg/kg/day	Ferrous sulphate
Name, Vasconcelos & Valzachi Rocha Maluf (2018)	Non-profit institution in the city of Poá, Brazil	20	1–13 years old with IDA	45 days	3 mg/kg/day	Iron Bisglycinate

All the included trials took place in developing countries. Five of the included trials were conducted in Turkey (Aycicek et al., 2014; Kavakli et al., 2004; Ozsurekci et al., 2015; Sozmen et al., 2003; Yasa, Agaoglu & Unuvar, 2011), while the others were each carried out in Brazil (Name, Vasconcelos & Valzachi Rocha Maluf, 2018), Israel (Jaber et al., 2010), and India (Bopche et al., 2009). Five trials involved outpatients from teaching institutions (Aycicek et al., 2014; Bopche et al., 2009; Kavakli et al., 2004; Ozsurekci et al., 2015; Sozmen et al., 2003; Yasa, Agaoglu & Unuvar, 2011), two trials involved participants from community centers (Jaber et al., 2010; Name, Vasconcelos & Valzachi Rocha Maluf, 2018), and one trial did not specify the setting for the recruitment of the participants (Sozmen et al., 2003).

The drug investigated in this review was IPC, and its efficacy and side effects were compared to ferrous iron. Six trials compared IPC to FS (Aycicek et al., 2014; Bopche et al., 2009; Kavakli et al., 2004; Ozsurekci et al., 2015; Sozmen et al., 2003; Yasa, Agaoglu & Unuvar, 2011), one trial to iron bisglycinate chelate (Name, Vasconcelos & Valzachi Rocha Maluf, 2018), and one trial to iron gluconate (Jaber et al., 2010). Both the intervention and control treatments were administered orally in all the included trials. However, none of the trials stated the exact content of each formulation used. The presence of other micronutrients, which may have influenced the results, was not clearly stated. Two trials used syrup formulations (Jaber et al., 2010; Yasa, Agaoglu & Unuvar, 2011), two trials used both tablets and syrups (Aycicek et al., 2014; Bopche et al., 2009), while four trials did not mention the actual type of formulation used (Kavakli et al., 2004; Name, Vasconcelos & Valzachi Rocha Maluf, 2018; Ozsurekci et al., 2015; Sozmen et al., 2003).

Furthermore, the dosage and duration of treatment were inconsistent across the trials. One trial on the prevention of IDA gave healthy infants from age four to six months 7.5mg/day, and 15 mg/day being administered from age six months to one year (Jaber et al., 2010). Among the seven trials on the treatment of IDA, one trial gave patients 3 mg/kg/day for 45 days (Name, Vasconcelos & Valzachi Rocha Maluf, 2018), one trial gave 5 mg/kg/day for one month (Aycicek et al., 2014), while another provided 5 mg/kg/day for four months (Yasa, Agaoglu & Unuvar, 2011). In four trials, 6 mg/kg/day was administered for different periods, namely, one month (Bopche et al., 2009), two months (Ozsurekci et al., 2015), and three months (Kavakli et al., 2004; Sozmen et al., 2003). Table 1 summarizes the intervention applied in each trial in detail.

We found that the wide variations in the dosage and duration of treatment were closely related to the choice of iron supplementation. We therefore decided to analyze the trials separately after categorizing the trials into three comparison groups. The first group compared IPC and iron gluconate for the prevention of IDA (Jaber et al., 2010), the second group compared IPC and bisglycinate chelate for the treatment of IDA (Name, Vasconcelos & Valzachi Rocha Maluf, 2018), and the third group compared IPC and FS for the treatment of IDA (Aycicek et al., 2014; Bopche et al., 2009; Kavakli et al., 2004; Ozsurekci et al., 2015; Sozmen et al., 2003; Yasa, Agaoglu & Unuvar, 2011).

### Outcomes

With respect to the primary outcomes, all the trials reported the final Hb level (Aycicek et al., 2014; Bopche et al., 2009; Jaber et al., 2010; Kavakli et al., 2004; Name, Vasconcelos & Valzachi Rocha Maluf, 2018; Ozsurekci et al., 2015; Sozmen et al., 2003; Yasa, Agaoglu & Unuvar, 2011), and five trials reported the ferritin level results (Aycicek et al., 2014; Jaber et al., 2010; Name, Vasconcelos & Valzachi Rocha Maluf, 2018; Sozmen et al., 2003; Yasa, Agaoglu & Unuvar, 2011). In terms of diagnosis, only one trial (Name, Vasconcelos & Valzachi Rocha Maluf, 2018) used age-standardized Hb and ferritin levels as per the WHO guidelines. The other trials use a variety of Hb cut-off points among the age groups. All except one (Jaber et al., 2010) trial declared that all the Hb levels was comparable between the intervention and control groups at baseline. By the end of each trial, six had successfully achieved normal Hb levels (Jaber et al., 2010; Kavakli et al., 2004; Name, Vasconcelos & Valzachi Rocha Maluf, 2018; Ozsurekci et al., 2015; Sozmen et al., 2003; Yasa, Agaoglu & Unuvar, 2011). Two trials had unresolved anemia with a mean Hb of less than 11 g/L at the end of the study (Aycicek et al., 2014; Bopche et al., 2009).

With regard to the secondary outcomes, four trials reported iron levels (Aycicek et al., 2014; Jaber et al., 2010; Sozmen et al., 2003; Yasa, Agaoglu & Unuvar, 2011), three trials reported both the MCV and MCH levels (Jaber et al., 2010; Name, Vasconcelos & Valzachi Rocha Maluf, 2018; Yasa, Agaoglu & Unuvar, 2011), and four trials reported gastrointestinal disturbances as side effects of the drugs (Aycicek et al., 2014; Bopche et al., 2009; Jaber et al., 2010; Yasa, Agaoglu & Unuvar, 2011). The gastrointestinal side effects that were reported include spitting or vomiting (Jaber et al., 2010), constipation (Jaber et al., 2010; Kavakli et al., 2004; Yasa, Agaoglu & Unuvar, 2011), diarrhea (Kavakli et al., 2004), and nausea or abdominal pain (Aycicek et al., 2014; Kavakli et al., 2004; Yasa, Agaoglu & Unuvar, 2011). One trial (Bopche et al., 2009) only mentioned gastrointestinal side effects in general. Tooth staining was reported in two trials, but this was not included in the meta-analysis (Jaber et al., 2010; Kavakli et al., 2004).

### Assessment of risk of bias

**Figure 2:**
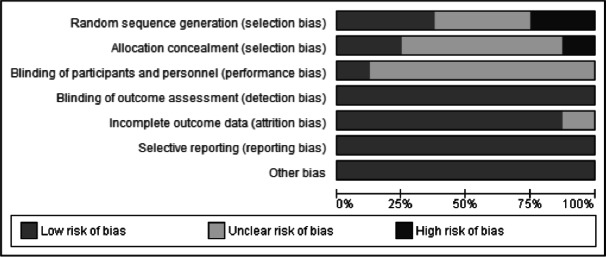
Risk of bias graph. Review authors’ judgments about each risk of bias item presented as percentages across all included studies.

**Figure 3:**
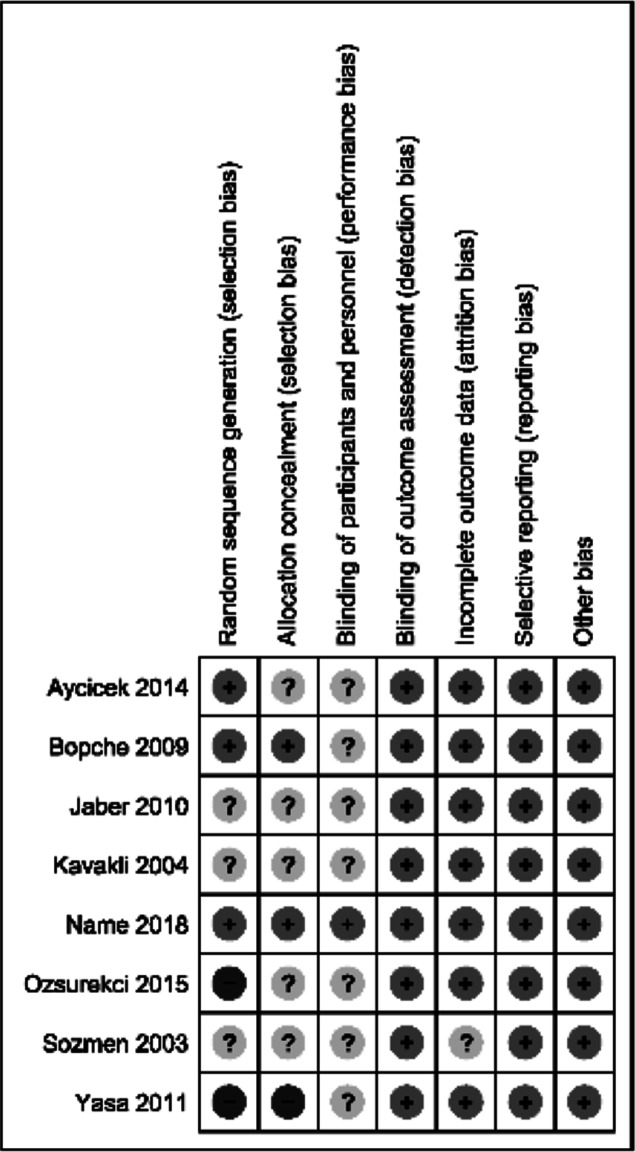
Risk of bias summary. Review authors’ judgements about each risk of bias item for each included study. (-) high risk of bias, (?) unclear risk of bias, (+) low risk of bias.

The assessment of the risk of bias is presented in Figs. 2 and 3. Three trials did not describe the method used to randomize the participants (Jaber et al., 2010; Kavakli et al., 2004; Sozmen et al., 2003). Two trials applied a simple randomization technique (Aycicek et al., 2014; Bopche et al., 2009). One trial used computer-generated random numbers (Name, Vasconcelos & Valzachi Rocha Maluf, 2018). Two trials had a high risk of randomization bias because the treatment allocation of newly recruited patients was alternated in a consecutive fashion (Ozsurekci et al., 2015) and on a weekly basis (Yasa, Agaoglu & Unuvar, 2011).

Proper allocation concealment and blinding in a study will produce better data validity and reliability, as well as reduce the risk of bias. However, allocation concealment was not mentioned in five trials (Aycicek et al., 2014; Jaber et al., 2010; Kavakli et al., 2004; Ozsurekci et al., 2015; Sozmen et al., 2003), and concealment was not performed in one trial (Yasa, Agaoglu & Unuvar, 2011). Two trials mentioned their methods of concealment: in one concealment was handled via a sealed envelope technique (Bopche et al., 2009), while in the other, an independent statistician was used (Name, Vasconcelos & Valzachi Rocha Maluf, 2018). Only one trial blinded the participants (Name, Vasconcelos & Valzachi Rocha Maluf, 2018). None of the other seven trials mentioned blinding of the participants.

All the trials reported the outcomes as specified in their objectives, and all trials analyzed the data according to the groups to which the participants were initially assigned. Three trials had between 10% and 46% data attrition due to loss to follow up (Bopche et al., 2009; Kavakli et al., 2004; Ozsurekci et al., 2015). One trial had up to 14% missing data because of loss to follow up or because some enrolled participants were later found to be ineligible (Jaber et al., 2010). One study excluded 15% of the participants for the improper use of the prescribed supplementation (Aycicek et al., 2014). One trial did not mention the reason for 32% missing outcomes data (Sozmen et al., 2003). Even though the attrition rate seemed high in percentage terms in all the trials, the number of missing participants did not result in a large difference in proportions between the intervention and control groups, and no baseline imbalances were identified.

### Iron supplementation for the prevention of IDA

#### Comparison between IPC and iron gluconate

Only one trial was available for this comparison (Jaber et al., 2010). For the primary outcomes, the IPC reported lower Hb level (MD −0.36, 95% CI −0.40 to −0.32; *P* < 0.001; one study, 105 participants, low certainty evidence) and lower ferritin level (MD −3.60, 95% CI −4.77 to −2.43; *P* < 0.001; one study, 105 participants, low certainty evidence) compared to iron gluconate (Figs. S2A and S2B). For the secondary outcomes, the IPC showed no difference in iron level compared to iron gluconate (MD −1.00, 95% CI −2.28 to 0.28; *P* = 0.130; one study, 105 participants, low certainty evidence) but lower MCV (MD −0.91, 95% CI −1.16 to −0.66; *P* < 0.001; one study, 105 participants, low certainty evidence) and MCH levels (MD −1.48, 95% CI −1.77 to −1.19; *P* < 0.001; one study, 105 participants, low certainty evidence) compared to iron gluconate (Figs. S2C, S2D and S2E). There was no difference in adverse events between the two groups (RR 0.81, 95% CI 0.33 to 2.00; *P* = 0.650; one study, 105 participants, low certainty evidence) (Fig. S2F).

### Iron supplementation for the treatment of IDA

#### Comparison between IPC and iron bisglycinate chelate

Only one trial was available for this comparison (Name, Vasconcelos & Valzachi Rocha Maluf, 2018). For the primary outcomes, the IPC showed no difference in Hb (MD 0.00, 95% CI −0.23 to 0.23; *P* = 1.000; one study, 20 participants, low certainty evidence) and ferritin level (MD −3.00, 95% CI −7.00 to 1.00; *P* = 0.140; one study, 20 participants, low certainty evidence) compared to iron bisglycinate chelate (Figs. S3A and S3B). For the secondary outcomes, the IPC reported higher MCV (MD 1.90, 95% CI 0.21 to 3.59; *P* = 0.030; one study, 20 participants, low certainty evidence) and MCH levels (MD 1.10, 95% CI 0.38 to 1.82; *P* = 0.003; one study, 20 participants, low certainty evidence) compared to iron bisglycinate chelate (Figs. S3C and S3D).

#### Comparison between IPC and ferrous sulphate (FS)

##### Primary outcomes.

**Figure 4:**
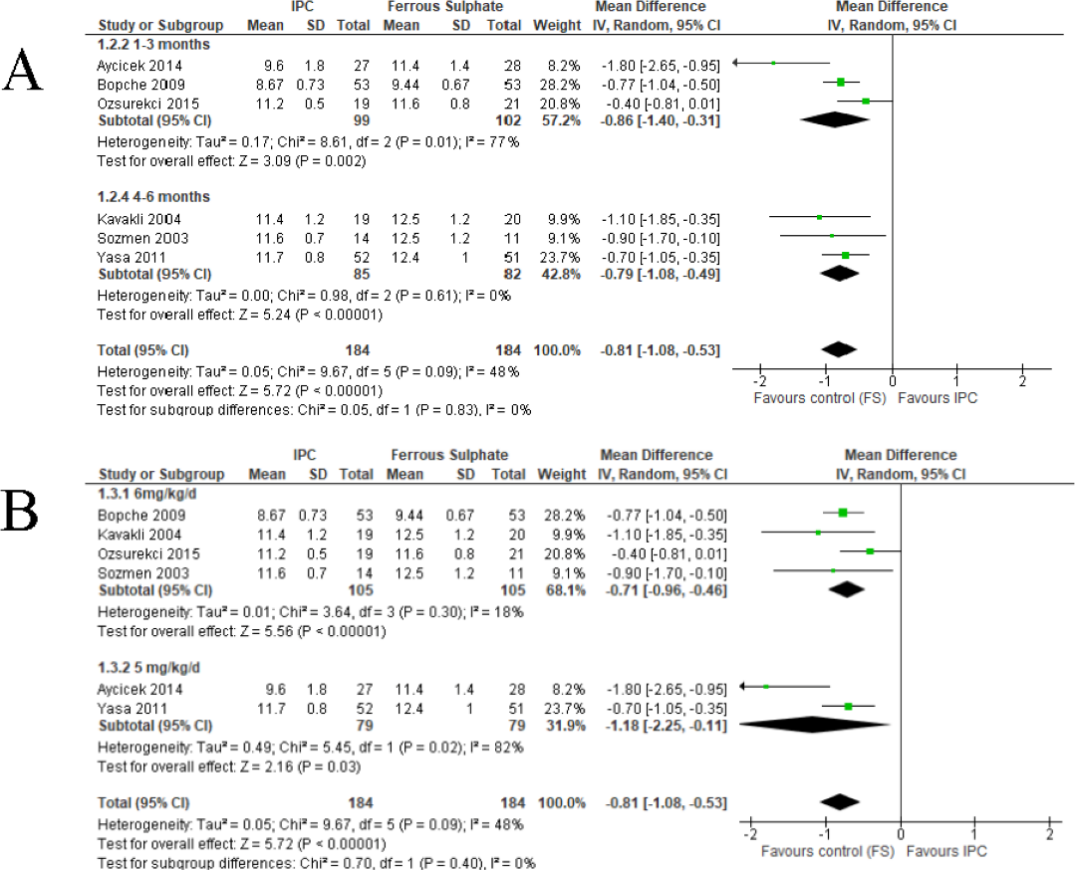
Forest plot comparing IPC with ferrous sulphate (FS) for the treatment of IDA in children, primary outcome 1: Hb level. (A) Subgroup analysis of Hb level according to duration of treatment. (B) Subgroup analysis of Hb level according to dosage of elemental iron.

Summary of the findings for comparison of iron polymaltose complex and FS is shown in Table 2. Six trials reported on Hb level for this comparison (Aycicek et al., 2014; Bopche et al., 2009; Kavakli et al., 2004; Ozsurekci et al., 2015; Sozmen et al., 2003; Yasa, Agaoglu & Unuvar, 2011). The IPC reported lower Hb level (MD −0.81, 95% CI −1.08 to −0.53, random-effects; I^2^ = 48%, *P* < 0.001; six studies, 368 participants; high certainty of evidence) compared to FS (Fig. 4 and Table 2). Subgroup analysis of Hb level by the duration of treatment were divided into two subgroups. Three trials which gave treatment over one to three months (Aycicek et al., 2014; Bopche et al., 2009; Ozsurekci et al., 2015) showed that IPC reported lower Hb level (MD −0.86, 95% CI −1.40 to −0.31, random-effects; I^2^ = 77%, *P* = 0.002; 3 studies, 201 participants) compared to FS. The other three trials which gave treatment over four to six months (Kavakli et al., 2004; Sozmen et al., 2003; Yasa, Agaoglu & Unuvar, 2011) showed lower Hb level for IPC (MD −0.79, 95% CI −1.08 to −0.49, random-effects; I^2^ 0%, *P* < 0.001; 3 studies, 167 participants) compared to FS (Fig. 4A).

**Table 2:** The GRADE quality assessment. Comparing IPC with ferrous sulphate for the treatment of IDA in children.

**Iron polymaltose complex (IPC) compared to ferrous sulphate (FS) for children with iron deficiency anaemia**						
**Patient or population:** children with iron deficiency anaemia						
**Setting:** outpatient or community clinic						
**Intervention:** iron polymaltose complex (IPC)						
**Comparison:** ferrous sulphate (FS)						
**Outcomes**	Anticipated absolute effects^*^(95% CI)		**Relative effect (95% CI)**	**No. of participants (studies)**	**Certainty of the evidence (GRADE)**	**Comments**
	**Risk with ferrous sulphate (FS)**	**Risk with iron polymaltose complex (IPC)**				
Hb level	The mean Hb level was 0	MD 0.81 lower (1.08 lower to 0.53 lower)	–	368 (6 RCTs)	⊕⊕⊕⊕ HIGH	Iron polymaltose complex (IPC) results in a slight reduction in Hb level compared to ferrous sulphate
Ferritin	The mean ferritin was 0	MD 21.24 lower (39.26 lower to 3.23 lower)	–	183 (3 RCTs)	⊕⊕⊕⊝ MODERATE ^1^	Iron polymaltose complex (IPC) likely results in a reduction in ferritin level compared to ferrous sulphate.
Iron level	The mean iron level was 0	MD 14.3 lower (33.03 lower to 4.43 higher)	–	183 (3 RCTs)	⊕⊕⊕⊝ MODERATE ^2^	Iron polymaltose complex (IPC) probably results in little to no difference in iron level compared to ferrous sulphate
MCV level	The mean MCV level was 0	MD 3.2 lower (5.35 lower to 1.05 lower)	–	103 (1 RCT)	⊕⊕⊝⊝ LOW ^34^	Iron polymaltose complex (IPC) may result in a slight reduction in MCV level compared to ferrous sulphate
MCH level	The mean MCH level was 0	MD 0.9 lower (1.85 lower to 0.05 higher)	–	103 (1 RCT)	⊕⊕⊝⊝ LOW ^34^	Iron polymaltose complex (IPC) may result in little to no difference in MCH level compared to ferrous sulphate
Adverse effects	Study population		RR 0.78 (0.47 to 1.31)	274 (3 RCTs)	⊕⊕⊕⊕HIGH	Iron polymaltose complex (IPC) results in no difference in adverse effects compared to ferrous sulphate
	212 per 1,000	165 per 1,000 (99 to 277)				

**Notes.**

* **The risk in the intervention group** (and its 95% confidence interval) is based on the assumed risk in the comparison group and the **relative effect** of the intervention (and its 95% CI).

CI Confidence interval RR Risk ratio

**GRADE Working Group grades of evidence**

**High certainty:** We are very confident that the true effect lies close to that of the estimate of the effect

**Moderate certainty:** We are moderately confident in the effect estimate: The true effect is likely to be close to the estimate of the effect, but there is a possibility that it is substantially different

**Low certainty:** Our confidence in the effect estimate is limited: The true effect may be substantially different from the estimate of the effect

**Very low certainty:** We have very little confidence in the effect estimate: The true effect is likely to be substantially different from the estimate of effect

**Explanations**

Substantial heterogeneity presence with minimal overlapping of confidence intervals (CI) across the included trials and I^2^ of 65% ( *P* = 0.06)

Substantial heterogeneity presence across the included trials with I^2^ of 57% ( *P* = 0.10)

Only one trial included. The trial has high risk of selection bias

Small sample size from one trial on 103 participants only

Subgroup analysis of Hb level by the dosage of elemental iron were divided into two groups. Four trials used 6 mg/kg/day of elemental irons (Bopche et al., 2009; Kavakli et al., 2004; Ozsurekci et al., 2015; Sozmen et al., 2003) showed that IPC reported lower Hb level (MD −0.71, 95% CI −0.96 to −0.46, random-effects; I^2^ = 18%, *P* < 0.001; 4 studies, 210 participants) compared to FS. Two trials gave 5 mg/kg/day of elemental iron (Aycicek et al., 2014; Yasa, Agaoglu & Unuvar, 2011) reported lower Hb level for IPC (MD −1.18, 95% CI −2.25 to −0.11, random-effects; I^2^ = 82%, *P* = 0.03; 2 studies, 158 participants) compared to FS (Fig. 4B).

**Figure 5:**
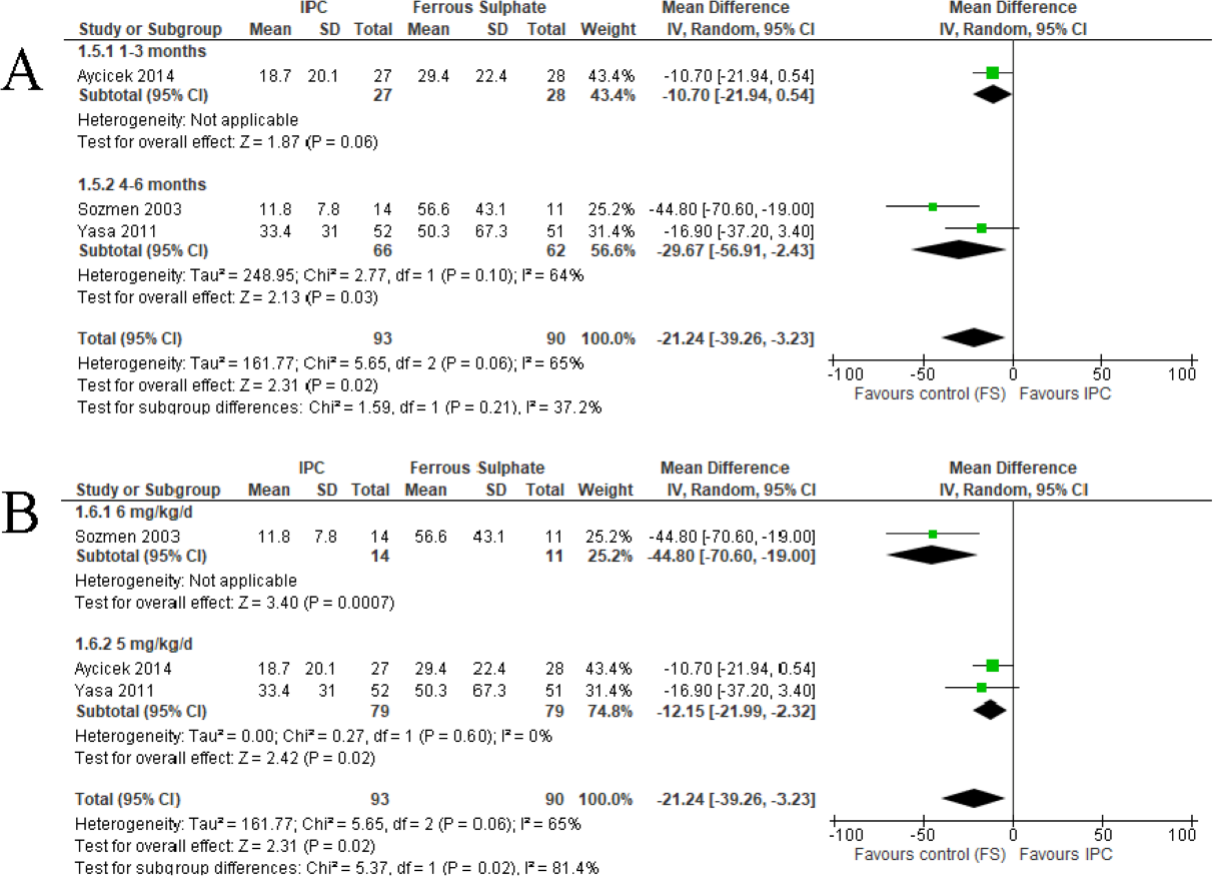
Forest plot comparing IPC and ferrous sulphate (FS) for the treatment of IDA in children, primary outcome 2: Ferritin level. (A) Subgroup analysis of ferritin level according to duration of treatment. (B) Subgroup analysis of ferritin level according to dosage of elemental iron.

For ferritin level, three trials (Aycicek et al., 2014; Sozmen et al., 2003; Yasa, Agaoglu & Unuvar, 2011) showed that IPC reported lower ferritin level (MD −21.24, 95% CI −39.26 to −3.23, random-effects; I^2^ = 65%, *P* = 0.020; 3 studies, 183 participants; moderate certainty of evidence) compared to FS (Fig. 5 and Table 2). Subgroup analysis of ferritin level by the duration of treatment showed the IPC reported lower ferritin level compared to FS. One trial (Aycicek et al., 2014) were included into one to three months duration of treatment (MD −10.70, 95% CI −21.94 to 0.54, random effects; *P* = 0.060; 1 study, 55 participants). Two trials (Sozmen et al., 2003; Yasa, Agaoglu & Unuvar, 2011) were included into four to six months duration of treatment (MD −29.67, 95% CI −56.91 to −2.43, random-effects; I^2^ = 64%, *P* = 0.020; 2 studies, 128 participants) (Fig. 5A).

Subgroup analysis of ferritin level by the dosage of elemental iron was divided into two groups. One trial (Sozmen et al., 2003) used 6 mg/kg/day of elemental iron reported lower ferritin level for IPC (MD −44.80, 95% CI −70.60 to −19.00, random effects; *p* < 0.001; 1 studies, 25 participants) compared to FS. Two trials (Aycicek et al., 2014; Yasa, Agaoglu & Unuvar, 2011) used 5 mg/kg/day of elemental iron showed similar result (MD −12.15, 95% CI −21.99 to −2.32, random-effects; I^2^ = 0%, *P* = 0.020; 2 studies, 158 participants) (Fig. 5B).

The sensitivity analysis did not result in a substantial change in the effect sizes and CI of trials with a high risk of selection bias (Ozsurekci et al., 2015; Yasa, Agaoglu & Unuvar, 2011). Subgroup analysis of the primary outcomes according to the timing of the consumption of the iron preparations were not performed in view of insufficient data. Five trials did not specify whether the iron preparations were consumed at mealtimes (Aycicek et al., 2014; Bopche et al., 2009; Kavakli et al., 2004; Sozmen et al., 2003; Yasa, Agaoglu & Unuvar, 2011), and only one trial mentioned that the iron preparations were given on an empty stomach (Ozsurekci et al., 2015).

#### Secondary outcomes

Three trials reported on the serum iron level (Aycicek et al., 2014; Sozmen et al., 2003; Yasa, Agaoglu & Unuvar, 2011). The IPC showed no difference in iron levels (MD −14.30, 95% CI −33.03 to 4.43, random effects; I^2^ = 57%, *P* = 0.130; 3 studies, 183 participants; moderate certainty of evidence) compared to FS (Fig. S1A and Table 2). Only one trial reported on the MCV and MCH level (Yasa, Agaoglu & Unuvar, 2011). IPC reported lower MCV level (MD −3.20, 95% CI −5.35 to −1.05, random-effects; *P* = 0.003; 1 study, 103 participants; low certainty of evidence) and no difference in MCH level (MD −0.90, 95% CI −1.85 to 0.05, random effects; *P* = 0.060; 1 study, 103 participants; low certainty of evidence) compared to FS (Figs. S1B and S1C and Table 2).

Three trials (Aycicek et al., 2014; Bopche et al., 2009; Yasa, Agaoglu & Unuvar, 2011) reported side effects in the form of gastrointestinal disturbances. The IPC showed no difference in occurrence of side effects (MD 0.78, 95% CI 0.47 to 1.31, random effects; I^2^ = 4%, *P* = 0.35; 3 studies, 274 participants; high certainty of evidence) compared to FS (Fig. S1D and Table 2).

## Discussion

IDA is the most dominant cause of anemia and anemia-related disability in most countries and populations. Standardized diagnoses according to age reduce the variations in population structure between countries and over time (Kassebaum, 2016). All countries have a significant prevalence rate (more than 10%), but it is higher in developing countries (28,704 per 100,000) than in developed countries (19,270 per 100,000) (Kassebaum, 2016).

Identifying IDA based on clinical symptoms can be challenging. Patients with mild to moderate anemia may be asymptomatic. Typical presentations, such as pallor, fatigue, dyspnea, and headache are very non-specific and documented in less than 50% of cases (Lopez et al., 2016). Other less common but debilitating implications of IDA includes impaired psychomotor and mental development, angina pectoris, congestive heart failure, as well as increased susceptibility to infections (Subramaniam & Girish, 2015). The effects of oral iron supplementation on the clinical outcomes in patients with IDA would therefore have a considerable impact on clinical practice.

This review was designed to include all RCTs addressing the effectiveness of oral IPC compared to oral ferrous preparations for the prevention and treatment of IDA in children. All four types of oral iron formulations included in this review showed positive effects on the hematological markers of anemia. However, in the comparison between IPC and ferrous sulphate for treatment of IDA in children, IPC resulted in lower Hb, ferritin and MCV levels compared to ferrous sulphate. The difference of 0.81 g/L of Hb levels and 21.24 µg/L of ferritin levels in the treatment of mild-to-moderate IDA in such short treatment periods seem clinically meaningful. However, there was unexplained moderate heterogeneity in the primary outcomes.

Performing a subgroup analysis according to different treatment durations and dosages failed to produce homogenous results. Thus, this lack of homogeneity is likely to have been caused by other factors. Subgroup analyses based on age group in line with age-standardized Hb levels, the severity of anemia, different generations of iron supplementation products, and the timing of consumption in relation to mealtimes (i.e., with a meal or on an empty stomach) were not carried out as these were inadequately reported.

The occurrence of gastrointestinal side effects such as spitting, vomiting, constipation, nausea and stomachache was comparable between IPC and ferrous sulphate. Other important side effects such as dyspepsia, alteration of the gastrointestinal microbiome, esophageal ulceration, and exacerbation of gastrointestinal inflammation (DeLoughery, 2019) were not addressed in this review as these side effects were not presented in the included trials. Consequently, subgroup analyses of each of the reported gastrointestinal side effects were not feasible given the lack of evidence.

Data comparing IPC with iron gluconate for the prevention of IDA and iron bisglycinate chelate for the treatment of IDA were very limited, and there was low certainty of evidence. The one study on the prevention of IDA (Jaber et al., 2010) had a small age range and represented a population younger than 12 months. Furthermore, the approach to IDA in infants younger than six months old remained uncertain as there are no established guidelines for diagnosing anemia in this age group (WHO, 2017). None of the trials presented data for IDA in neonates.

The quality of the evidence in this review varied from low to high. We graded the quality of the evidence as high for Hb and moderate for ferritin. The quality of evidence for the secondary outcomes ranged from low to moderate and high. The outcomes were primarily downgraded due to study limitations, high heterogeneity, and a small sample size. Most of the trials had a low or unclear risk of bias in most domains. The majority of the trials did not explain either the method of allocation concealment or the blinding of participants, personnel, and outcome assessments. However, we considered that all the trials had a low risk of detection bias given that the reported laboratory results were from blood samples.

Attrition bias was considered high in percentage terms in most of the trials (Fig. 3). However, in all the trials, the number of missing participants was balanced between the IPC and ferrous iron groups. There was no evidence of selective reporting bias. We attempted to minimize potential biases in the review process by having two review authors assess the trials’ eligibility for inclusion, carry out the data extraction and assess the risk of bias. We also performed grey literature searches by looking at the reference lists of all the related studies, searching multiple databases, and exploring unpublished papers such as conference proceedings, dissertations and theses.

Two published meta-analyses investigated the effectiveness and tolerability of ferrous sulphate in patients with IDA (Toblli & Brignoli, 2007; Tolkien et al., 2015). However, one review compared the tolerability of FS to a placebo (Tolkien et al., 2015). This review explored the gastrointestinal side effects in adult patients and confirmed that FS significantly increased the occurrence of gastrointestinal side effects compared to the placebo at any dose. Another systematic review studied the efficacy of IPC compared to FS in the treatment of IDA for all populations (Toblli & Brignoli, 2007). However, this review excluded pediatric trials from the meta-analysis because the groups were not comparable at baseline. The same review reported that the dropout rates were similar in the IPC and FS groups in the trials for both adults and children. However, the adverse drug reactions were significantly less frequent among the adults and children treated with IPC.

If further studies were to be conducted in the future, a number of limitations would need to be considered. First, the study population should represent age-standardized Hb levels (Kassebaum, 2016; WHO, 2017). This is because the prevalence, diagnosis and prognosis of IDA in children differs according to age group, sex, and anemia severity at diagnosis. Second, we recommend that trial authors use standardized elemental iron dosages, regimes and consumption timing in order to produce more homogenous outcomes (Camaschella, 2017; WHO, 2016). Disclosure of the compound and the presence of other micronutrients in each supplement that may affect the outcome is also essential (Das et al., 2019). Third, current literature does not evaluate the potency of IPC and ferrous iron in terms of improvements in relevant clinical outcomes, or the cost-effectiveness of each oral iron supplement.

## Conclusions

The current evidence showed that the difference between oral IPC and oral FS in improving hematological parameters was clinically meaningful, with FS being superior to IPC.There was no difference in the occurrence of gastrointestinal side effects between the IPC and FS groups. Owing to the limited number of studies in each subgroup, we were unable to draw definite conclusions from various subgroup analyses. Due to the paucity of good quality trials comparing IPC with iron gluconate and iron bisglycinate chelate, the effectiveness of these iron supplementations in the prevention and treatment of IDA remains unclear. High-quality trials with large sample sizes, that assessing both hematological and clinical outcomes are required.

##  Supplemental Information

10.7717/peerj.10527/supp-1 Supplemental Information 1 PRISMA checklist

10.7717/peerj.10527/supp-2 Figure S1 Forest plot of comparing IPC with ferrous sulphate (FS) for the treatment of IDA in children, secondary outcomes (A) Iron level. (B) MCV level. (C) MCH level. (D) Adverse effects (gastrointestinal disturbances).

10.7717/peerj.10527/supp-3 Figure S2 Forest plot comparing IPC with iron gluconate for the prevention of IDA (A) Primary outcome: Hb level. (B) Primary outcome: Ferritin level. (C) Secondary outcome :Iron level. (D) Secondary outcome: MCV level. (E) Secondary outcome: MCH level. (F) Secondary outcome: Adverse effects.

10.7717/peerj.10527/supp-4 Figure S3 Forest plot comparing IPC with iron bisglycinate chelate for the treatment of IDA (A) Primary outcome: Hb level. (B) Primary outcome: Ferritin level. (C) Secondary outcome: MCV level. (D) Secondary outcome: MCH level.

10.7717/peerj.10527/supp-5 Appendix S1 Search strategy

10.7717/peerj.10527/supp-6 Appendix S2 Review protocol **PROSPERO registration number: CRD42019145020**

10.7717/peerj.10527/supp-7 Appendix S3 Data extraction form A standardized form used to extract important data from the included trial. Randomized control trial 1: 2003 Sozmen.

10.7717/peerj.10527/supp-8 Appendix S4 Data extraction form A standardized form used to extract important data from the included trial. Randomized control trial 2: 2004 Kavakli.

10.7717/peerj.10527/supp-9 Appendix S5 Data extraction form A standardized form used to extract important data from the included trial. Randomized control trial 3: 2009 Bopche.

10.7717/peerj.10527/supp-10 Appendix S6 Data extraction form A standardized form used to extract important data from the included trial. Randomized control trial 4: 2010 Jaber.

10.7717/peerj.10527/supp-11 Appendix S7 Data extraction form A standardized form used to extract important data from the included trial. Randomized control trial 5: 2011 Yasa.

10.7717/peerj.10527/supp-12 Appendix S8 Data extraction form A standardized form used to extract important data from the included trial. Randomized control trial 6: 2014 Aycicek.

10.7717/peerj.10527/supp-13 Appendix S9 Data extraction form A standardized form used to extract important data from the included trial. Randomized control trial 7: 2015 Ozsurekci.

10.7717/peerj.10527/supp-14 Appendix S10 Data extraction form A standardized form used to extract important data from the included trial. Randomized control trial 8: 2018 Name.

10.7717/peerj.10527/supp-15 File S1 The rationale and significance for conducting this systematic review and meta-analysis

## Additional Information and Declarations

### Data Availability

The following information was supplied regarding data availability: The raw data, including the data extraction forms which were used to extract information from each included trials, are available in the Supplemental Files.
