# Supplementary material for: Effectiveness of iron polymaltose complex in treatment and prevention of iron deficiency anemia in children: a systematic review and meta-analysis
Source: PeerJ. 2021 Jan 13;9:e10527. doi: 10.7717/peerj.10527 (PMC7811280; doi:10.7717/peerj.10527)
Supplement: Supplemental Information 1 [file peerj-09-10527-s001.doc]

| **Section/topic** | **#** | **Checklist item** | **Reported on page #** | **Quoted text(s) from the manuscript** |
| --- | --- | --- | --- | --- |
| **TITLE** | | |  |  |
| Title | 1 | Identify the report as a systematic review, meta-analysis, or both. | 1 | *“Effectiveness of iron polymaltose complex in treatment and prevention of iron deficiency anemia in children: A systematic review and meta-analysis”* |
| **ABSTRACT** | | |  |  |
| Structured summary | 2 | Provide a structured summary including, as applicable: background; objectives; data sources; study eligibility criteria, participants, and interventions; study appraisal and synthesis methods; results; limitations; conclusions and implications of key findings; systematic review registration number. | 2-3 | ***Background: “****Iron deficiency anemia (IDA) is commonly treated with iron formulations. Despite the expanding acceptance of iron polymaltose complex (IPC) among clinicians, there is sparse and contradictory evidence regarding its efficacy in the management of IDA in children.”*  ***Objectives:*** *“This systematic review and meta-analysis aimed to assess the effectiveness of IPC in the treatment and prevention of IDA in children.”*  ***Data sources****: “We searched the Cochrane Central Register of Controlled Trials, MEDLINE and Epistemonikos”*  ***Study eligibility criteria, pariticipants, and intervention:*** *“all randomized control trials (RCTs) comparing oral IPC with standard oral iron supplementation for the treatment or prevention of IDA in children.”*  ***Study appraisal and synthesis method: “****We independently screened the titles and abstracts of identified trials before the full text of relevant trials was evaluated for eligibility. We then independently extracted data on the methods, interventions, outcomes, and risk of bias from the included trials. A random-effects model was used to estimate the risk ratios and mean differences with 95% confidence intervals.”*  ***Results.*** *“Eight trials comprising 493 randomized patients were included and analyzed using three comparison groups. The comparison group of which was used to evaluate IPC and ferrous sulphate (FS) for treatment of IDA showed that IPC is less effective in increasing Hb (MD -0.81, 95% CI -1.08 to -0.53; I² = 48%, P < 0.001; six studies, 368 participants; high certainty of evidence), ferritin (MD -0.81, 95% CI -1.08 to -0.53; I² = 48%, P < 0.001; six studies, 368 participants; high certainty of evidence) and MCV levels (MD -3.20, 95% CI -5.35 to -1.05; P = 0.003; one study, 103 participants; low certainty of evidence). There was no difference in the occurrence of side effects between IPC and FS group (MD 0.78, 95% CI 0.47 to 1.31; I² = 4%, P = 0.35; three studies, 274 participants; high certainty of evidence)”*  ***Conclusions and implications:*** *“The current limited evidence showed that the difference between oral IPC and oral FS in improving hematological parameters was trivial, with FS being marginally superior to IPC in the treatment of IDA in children. There was no difference in the occurrence of gastrointestinal side effects between the IPC and FS groups. Due to the paucity of good quality trials comparing IPC with iron gluconate and iron bisglycinate chelate, the effectiveness of these iron supplements in the prevention and treatment of IDA remains unclear.”*  ***Limitations:*** *“Adequately powered and high-quality trials with large sample sizes that assess both hematological and clinical outcomes are required.”*  *“PROSPERO registration number: CRD42019145020”* |
| **INTRODUCTION** | | |  |  |
| Rationale | 3 | Describe the rationale for the review in the context of what is already known. | 4 | *“Evidence has shown that the bioavailability of ferrous iron and IPC is comparable (Jacobs et al. 2000). However, several contradictory studies have made the efficacy of IPC a topic of debate (Geisser 2007). A meta-analysis in adult populations showed that there was no difference in Hb levels following treatment for IDA with either FS or IPC, but IPC was more tolerable (Toblli & Brignoli 2007). Similar results were reported the for treatment of IDA in a pregnant population (Ortiz et al. 2011). However, homogenous evidence on IPC for treatment and prevention of IDA in pediatric populations is not well established.”* |
| Objectives | 4 | Provide an explicit statement of questions being addressed with reference to participants, interventions, comparisons, outcomes, and study design (PICOS). | 4 | *“The objectives of this study were therefore to determine the efficacy and safety of oral iron IPC for the prevention and treatment of IDA in infants, children, and adolescents. The aim was to provide high-quality evidence comparing oral IPC and oral ferrous iron commonly used in routine practice.”* |
| **METHODS** | | |  |  |
| Protocol and registration | 5 | Indicate if a review protocol exists, if and where it can be accessed (e.g., Web address), and, if available, provide registration information including registration number. | 4 | *“Our systematic review was conducted according to the protocol previously published in the PROSPERO register (Registration no: CRD42019145020).”* |
| Eligibility criteria | 6 | Specify study characteristics (e.g., PICOS, length of follow-up) and report characteristics (e.g., years considered, language, publication status) used as criteria for eligibility, giving rationale. | 4 | *“We considered randomized controlled trials (RCTs) comparing oral IPC with iron salts for the prevention or treatment of IDA. We excluded cross-over trials because the primary outcomes are considered irreversible. The participants included children of both sexes and of any ethnicity. The interventions were oral IPC supplementation in either a preventative or treatment dosage. The comparators were selected based on the availability of comparative studies conducted against IPC.”*  *“We did not impose any exclusion criteria for the trials aside from language restrictions to maximize the number of included trials. Only English language publications were included given the limitations inherent to translating non-English papers.”* |
| Information sources | 7 | Describe all information sources (e.g., databases with dates of coverage, contact with study authors to identify additional studies) in the search and date last searched. | 5 | *“We searched the Cochrane Central Register of Controlled Trials, MEDLINE, and Epistemonikos from inception to July 2019.”*  *“We checked the reference list of the identified RCTs and review articles to find unpublished trials or trials not identified by the electronic searches. We also searched for ongoing trials through the WHO International Clinical Trials Registry Platform and ClinicalTrials.gov.”* |
| Search | 8 | Present full electronic search strategy for at least one database, including any limits used, such that it could be repeated. | 5 | *“We used the text words “iron deficiency anaemia”, “iron polymaltose complex” and “children” as well as Boolean operators like AND, OR, truncation, and wildcards for variations in words (Supplementary Appendix 1).”* |
| Study selection | 9 | State the process for selecting studies (i.e., screening, eligibility, included in systematic review, and, if applicable, included in the meta-analysis). | 5 | *“Two authors (RRMR, NMN) independently screened all the titles and abstracts identified through the implementation of the search strategy to determine the trials for further assessment. The full text of all trials that could potentially be included were screened and evaluated according to the eligibility criteria. Any reasons for exclusion were documented. Conflicts between the review authors were resolved by discussion. The study flow diagram (Fig. 1) mapped out the number of records identified, screened, included, and excluded (with reasons).”* |
| Data collection process | 10 | Describe method of data extraction from reports (e.g., piloted forms, independently, in duplicate) and any processes for obtaining and confirming data from investigators. | 5 | *“Using the data extraction form, the review authors (RRMR, NMN) independently extracted the key characteristics of the trials (study setting), the participants’ characteristics (age, sex, ethnicity, severity of IDA at baseline), the methodology (number of patients randomized and analyzed, duration of follow up, inclusion and exclusion criteria), the description of the intervention (preparation, dosage, duration), and the outcomes.”* |
| Data items | 11 | List and define all variables for which data were sought (e.g., PICOS, funding sources) and any assumptions and simplifications made. | 5 | *“The predefined primary outcomes included the level of Hb in g/L and serum ferritin in ng/mL or mcg/L at the end of treatment. These values were taken from the results of the full blood count and iron studies using venous blood samples that were sent to the respective laboratories and tested using the applicable local setting. The secondary outcomes were serum iron level (mcg/dL), MCV (fL), MCH (pg) and the occurrence of gastrointestinal disturbances (abdominal pain, nausea, vomiting, diarrhea and constipation) at any time during the study period after the participants had been randomized into the intervention and control groups.”* |
| Risk of bias in individual studies | 12 | Describe methods used for assessing risk of bias of individual studies (including specification of whether this was done at the study or outcome level), and how this information is to be used in any data synthesis. | 5,6 | *“All the included studies were assessed for risk of bias as outlined in the Cochrane Handbook of Systematic Reviews of Interventions (Higgins et al. 2019). Two review authors (RRMR, NMN) independently assessed each trial based on random sequence generation, allocation concealment, the blinding of participants and personnel, the blinding of outcome assessors, the completeness of the outcome data, the selectivity of outcome reporting, and other forms of bias. We categorized the risk of bias as low, unclear, or high.”* |
| Summary measures | 13 | State the principal summary measures (e.g., risk ratio, difference in means). | 6 | *“All the statistical analyses were carried out using Review Manager software (Review Manager 2011). For all the included trials with continuous outcomes, we calculated the mean differences (MDs) with 95% confidence intervals (CI), and for those with dichotomous outcomes, we presented the results as a summary of the risk ratios (RR). We further reported the results using the random effects model”* |
| Synthesis of results | 14 | Describe the methods of handling data and combining results of studies, if done, including measures of consistency (e.g., I^2^) for each meta-analysis. | 6 | *“We pooled these measures in the meta-analyses and drew forest plots.”*  *“We assessed for obvious heterogeneity by comparing the populations, interventions, comparators, and outcomes. We then assessed the statistical heterogeneity utilizing the I2 statistic (Higgins et al. 2019).”* |

Page 1 of 2

| **Section/topic** | **#** | **Checklist item** | **Reported on page #** | **Quoted text(s) from the manuscript** |
| --- | --- | --- | --- | --- |
| Risk of bias across studies | 15 | Specify any assessment of risk of bias that may affect the cumulative evidence (e.g., publication bias, selective reporting within studies). | 6 | *“Two review authors (RRMR, NMN) independently assessed each trial based on random sequence generation, allocation concealment, the blinding of participants and personnel, the blinding of outcome assessors, the completeness of the outcome data, the selectivity of outcome reporting, and other forms of bias.”* |
| Additional analyses | 16 | Describe methods of additional analyses (e.g., sensitivity or subgroup analyses, meta-regression), if done, indicating which were pre-specified. | 6 | *“We planned to carry out subgroup analyses according to the age of the children, the duration of treatment, the dose of elemental iron, and whether it was used for the prevention or treatment of IDA. We performed a sensitivity analysis to investigate the impact of risk of bias for sequence generation and allocation concealment on the included trials.”* |
| **RESULTS** | | |  |  |
| Study selection | 17 | Give numbers of studies screened, assessed for eligibility, and included in the review, with reasons for exclusions at each stage, ideally with a flow diagram. | 6, 7 | *“The electronic searches from inception until July 2019 retrieved a total of 69 records and three ongoing trials from other sources. After removing duplicate records, we screened 52 records and excluded 34 that did not meet the eligibility criteria. Of the remaining18 trials, six were excluded due to the inaccessibility of the English full text (Amaral et al. 2012; Arvas & Gur 2000; Borbolla et al. 2000; Haliotis & Papanastasiou 1998; Murahovschi et al. 1987; Schmidt et al. 1985).A further four trials were excluded because one did not fulfil the eligibility criteria for the study outcomes (Sheikh et al. 2017), one used intravenous IPC instead of oral IPC (Akarsu et al. 2013) and two trials did not compare IPC with a ferrous formulation (Afzal et al. 2009; Prasetyani et al. 2017). There were three records related to ongoing trials; one trial was using oral IPC (NCT 2014) and two trials were using a different type of ferric iron and did not meet the eligibility criteria for this review (NCT 2017; NCT 2018). Figure 1 summarizes the results of the search strategy.”* |
| Study characteristics | 18 | For each study, present characteristics for which data were extracted (e.g., study size, PICOS, follow-up period) and provide the citations. | 7,8 | *“The eight trials included 493 participants. Only one trial was designed for anemia prevention in healthy infants (Jaber et al. 2010), and it was also the only trial with a population age below six months. The remaining seven trials focused on the treatment of children with IDA (Aycicek et al. 2014; Bopche et al. 2009; Kavakli et al. 2004; Name et al. 2018; Ozsurekci et al. 2015; Sozmen et al. 2003; Yasa et al. 2011). Table 1 summarized the characteristics of the included trials.”*  *“The drug investigated in this review was IPC, and its efficacy and side effects were compared to ferrous iron. Six trials compared IPC to FS (Aycicek et al. 2014; Bopche et al. 2009; Kavakli et al. 2004; Ozsurekci et al. 2015; Sozmen et al. 2003; Yasa et al. 2011), one trial to iron bisglycinate chelate (Name et al. 2018), and one trial to iron gluconate (Jaber et al. 2010).”*  *“ One trial on the prevention of IDA gave healthy infants from age four to six months 7.5mg/day and 15 mg/day being administered from age six months to one year (Jaber et al. 2010). Among the seven trials on the treatment of IDA, one trial gave patients 3 mg/kg/day for 45 days (Name et al. 2018), one trial gave 5 mg/kg/day for one month (Aycicek et al. 2014), while another provided 5 mg/kg/day for four months (Yasa et al. 2011). in four trials, 6 mg/kg/day was administered for different periods, namely, one month (Bopche et al. 2009), two months (Ozsurekci et al. 2015), and three months (Kavakli et al. 2004; Sozmen et al. 2003). Table 1 summarizes the intervention applied in each trial in detail.”* |
| Risk of bias within studies | 19 | Present data on risk of bias of each study and, if available, any outcome level assessment (see item 12). | 8,9 | *“The assessment of the risk of bias is presented in Fig. 2 and Fig. 3.”*  *“All the trials reported the outcomes as specified in their objectives, and all trials analyzed the data according to the groups to which the participants were initially assigned.”* |
| Results of individual studies | 20 | For all outcomes considered (benefits or harms), present, for each study: (a) simple summary data for each intervention group (b) effect estimates and confidence intervals, ideally with a forest plot. | 9-11 | *“Only one trial was available for this comparison (Jaber et al. 2010). For the primary outcomes, the IPC reported lower Hb level (MD -0.36, 95% CI -0.40 to -0.32; P < 0.001; one study, 105 participants, low certainty evidence) and lower ferritin level (MD -3.60, 95% CI -4.77 to -2.43; P <0.001; one study, 105 participants, low certainty evidence) compared to iron gluconate (Fig. S2A and S2B). For the secondary outcomes, the IPC showed no difference in iron level compared to iron gluconate (MD -1.00, 95% CI -2.28 to 0.28; P = 0.130; one study, 105 participants, low certainty evidence) but lower MCV (MD -0.91, 95% CI -1.16 to -0.66; P <0.001; one study, 105 participants, low certainty evidence) and MCH levels (MD -1.48, 95% CI -1.77 to -1.19; P <0.001; one study, 105 participants, low certainty evidence) compared to iron gluconate (Fig. S2C, S2D and S2E). There was no difference in adverse events between the two groups (RR 0.81, 95% CI 0.33 to 2.00; P = 0.650; one study, 105 participants, low certainty evidence) (Fig. S2F).”*  *“Only one trial was available for this comparison (Name et al. 2018). For the primary outcomes, the IPC showed no difference in Hb (MD 0.00, 95% CI -0.23 to 0.23; P = 1.000; one study, 20 participants, low certainty evidence) and ferritin level (MD -3.00, 95% CI -7.00 to 1.00; P = 0.140; one study, 20 participants, low certainty evidence) compared to iron bisglycinate chelate (Fig. S3A and S3B). For the secondary outcomes, the IPC reported higher MCV (MD 1.90, 95% CI 0.21 to 3.59; P = 0.030; one study, 20 participants, low certainty evidence) and MCH levels (MD 1.10, 95% CI 0.38 to 1.82; P = 0.003; one study, 20 participants, low certainty evidence) compared to iron bisglycinate chelate (Fig. S3C and S3D).”*  *“Summary of the findings for comparison of iron polymaltose complex and FS is shown in Table 2. Six trials reported on Hb level for this comparison (Aycicek et al. 2014; Bopche et al. 2009; Kavakli et al. 2004; Ozsurekci et al. 2015; Sozmen et al. 2003; Yasa et al. 2011). The IPC reported lower Hb level (MD -0.81, 95% CI -1.08 to -0.53, random-effects; I² = 48%, P < 0.001; six studies, 368 participants; high certainty of evidence) compared to FS (Fig. 4 and Table 2). Subgroup analysis of Hb level by the duration of treatment were divided into two subgroups. Three trials which gave treatment over one to three months (Aycicek et al. 2014; Bopche et al. 2009; Ozsurekci et al. 2015) showed that IPC reported lower Hb level (MD -0.86, 95% CI -1.40 to -0.31, random-effects; I² = 77%, P = 0.002; 3 studies, 201 participants) compared to FS. The other three trials which gave treatment over four to six months (Kavakli et al. 2004; Sozmen et al. 2003; Yasa et al. 2011) showed lower Hb level for IPC (MD -0.79, 95% CI -1.08 to -0.49, random-effects; I² 0%, P < 0.001; 3 studies, 167 participants) compared to FS (Fig. 4A)”* |
| Synthesis of results | 21 | Present results of each meta-analysis done, including confidence intervals and measures of consistency. | 9-11 |
| Risk of bias across studies | 22 | Present results of any assessment of risk of bias across studies (see Item 15). | 9 | *“All the trials reported the outcomes as specified in their objectives, and all trials analyzed the data according to the groups to which the participants were initially assigned.”* |
| Additional analysis | 23 | Give results of additional analyses, if done (e.g., sensitivity or subgroup analyses, meta-regression [see Item 16]). | 10,11 | *“Subgroup analysis of ferritin level by the dosage of elemental iron was divided into two groups. One trial (Sozmen et al. 2003) used 6 mg/kg/day of elemental iron reported lower ferritin level for IPC (MD -44.80, 95% CI -70.60 to -19.00, random effects; p <0.001; 1 studies, 25 participants) compared to FS. Two trials (Aycicek et al. 2014; Yasa et al. 2011) used 5 mg/kg/day of elemental iron showed similar result (MD -12.15, 95% CI -21.99 to -2.32, random-effects; I² = 0%, P = 0.020; 2 studies, 158 participants) (Fig. 5B).*  *The sensitivity analysis did not result in a substantial change in the effect sizes and CI of trials with a high risk of selection bias (Ozsurekci et al. 2015; Yasa et al. 2011). Subgroup analysis of the primary outcomes according to the timing of the consumption of the iron preparations were not performed in view of insufficient data. Five trials did not specify whether the iron preparations were consumed at mealtimes (Aycicek et al. 2014; Bopche et al. 2009; Kavakli et al. 2004; Sozmen et al. 2003; Yasa et al. 2011), and only one trial mentioned that the iron preparations were given on an empty stomach (Ozsurekci et al. 2015).”* |
| **DISCUSSION** | | |  |  |
| Summary of evidence | 24 | Summarize the main findings including the strength of evidence for each main outcome; consider their relevance to key groups (e.g., healthcare providers, users, and policy makers). | 12 | *“All four types of oral iron formulations included in this review showed positive effects on the hematological markers of anemia. However, in the comparison between IPC and ferrous sulphate for treatment of IDA in children, IPC resulted in lower Hb, ferritin and MCV levels compared to ferrous sulphate. Notwithstanding, the difference in Hb levels was trivial, and there was unexplained moderate heterogeneity in the primary outcomes.”*  *“The occurrence of gastrointestinal side effects such as spitting, vomiting, constipation, nausea and stomachache was comparable between IPC and ferrous sulphate.”* |
| Limitations | 25 | Discuss limitations at study and outcome level (e.g., risk of bias), and at review-level (e.g., incomplete retrieval of identified research, reporting bias). | 12, 13 | *“Performing a subgroup analysis according to different treatment durations and dosages failed to produce homogenous results.”*  *“Subgroup analyses based on age group in line with age-standardized Hb levels, the severity of anemia, different generations of iron supplementation products, and the timing of consumption in relation to mealtimes (i.e. with a meal or on an empty stomach) were not carried out as these were inadequately reported.”*  *“Other important side effects such as dyspepsia, alteration of the gastrointestinal microbiome, esophageal ulceration, and exacerbation of gastrointestinal inflammation (DeLoughery 2019) were not addressed in this review as these side effects were not presented in the included trials. Consequently, subgroup analyses of each of the reported gastrointestinal side effects were not feasible given the lack of evidence.”*  *“Data comparing IPC with iron gluconate for the prevention of IDA and iron bisglycinate chelate for the treatment of IDA were very limited, and there was low certainty of evidence.”*    *“The quality of the evidence in this review varied from low to high.”*  *“The outcomes were primarily downgraded due to study limitations, high heterogeneity, and a small sample size. Most of the trials had a low or unclear risk of bias in most domains. The majority of the trials did not explain either the method of allocation concealment or the blinding of participants, personnel, and outcome assessments.”*  *“Attrition bias was considered high in percentage terms in most of the trials (Fig. 3). However, in all the trials, the number of missing participants was balanced between the IPC and ferrous iron groups.”* |
| Conclusions | 26 | Provide a general interpretation of the results in the context of other evidence, and implications for future research. | 13 | *“The current limited evidence showed that the difference between oral IPC and oral FS in improving hematological parameters was trivial, with FS being marginally superior to IPC. There was no difference in the occurrence of gastrointestinal side effects between the IPC and FS groups. Owing to the limited number of studies in each subgroup, we were unable to draw definite conclusions from various subgroup analyses. Due to the paucity of good quality trials comparing IPC with iron gluconate and iron bisglycinate chelate, the effectiveness of these iron supplements in the prevention and treatment of IDA remains unclear. High-quality trials with large sample sizes, that assessing both hematological and clinical outcomes are required.”* |
| **FUNDING** | | |  |  |
| Funding | 27 | Describe sources of funding for the systematic review and other support (e.g., supply of data); role of funders for the systematic review. | - | This part has been removed from the manuscript in order to fulfill the journal’s format as stated in “PeerJ Resubmission Checklist”. |

*From:*  Moher D, Liberati A, Tetzlaff J, Altman DG, The PRISMA Group (2009). Preferred Reporting Items for Systematic Reviews and Meta-Analyses: The PRISMA Statement. PLoS Med 6(7): e1000097. doi:10.1371/journal.pmed1000097

For more information, visit: **www.prisma-statement.org**.

Page 2 of 2
