# Supplementary figures and images for: Effectiveness of iron polymaltose complex in treatment and prevention of iron deficiency anemia in children: a systematic review and meta-analysis
Source: PeerJ. 2021 Jan 13;9:e10527. doi: 10.7717/peerj.10527 (PMC7811280; doi:10.7717/peerj.10527)

A

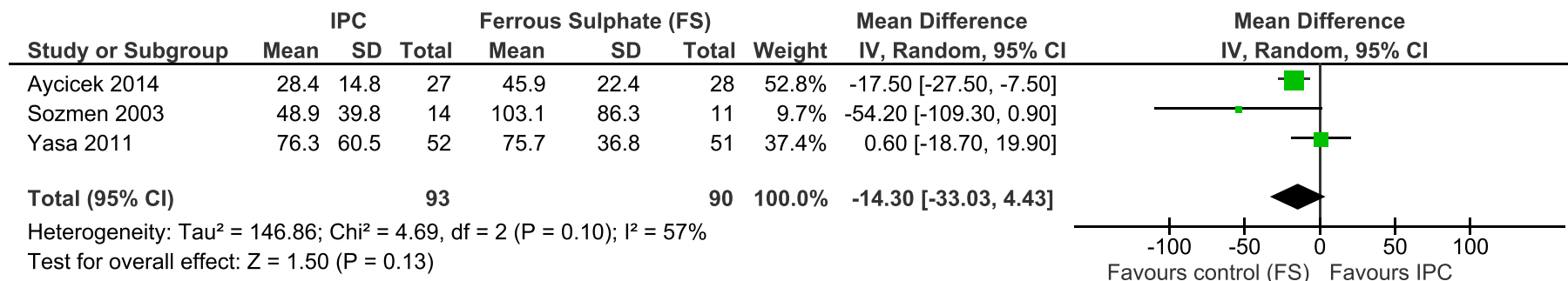

B

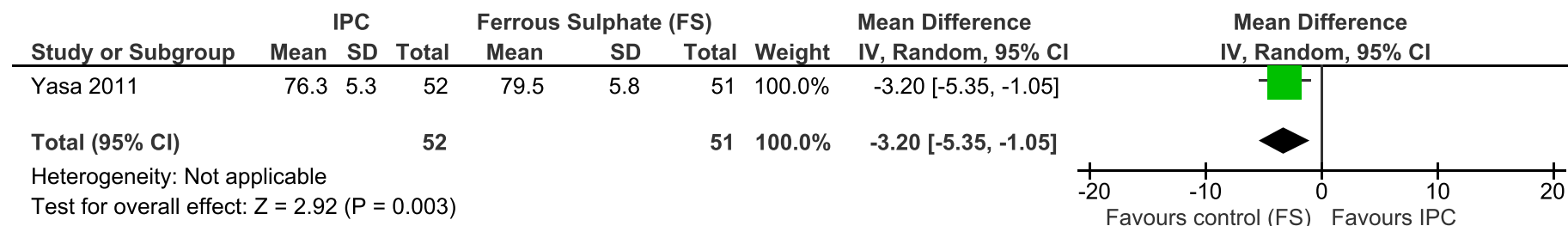

C

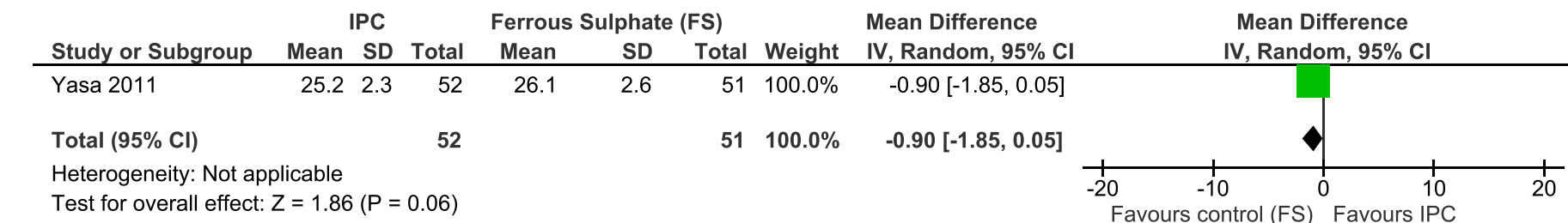

D

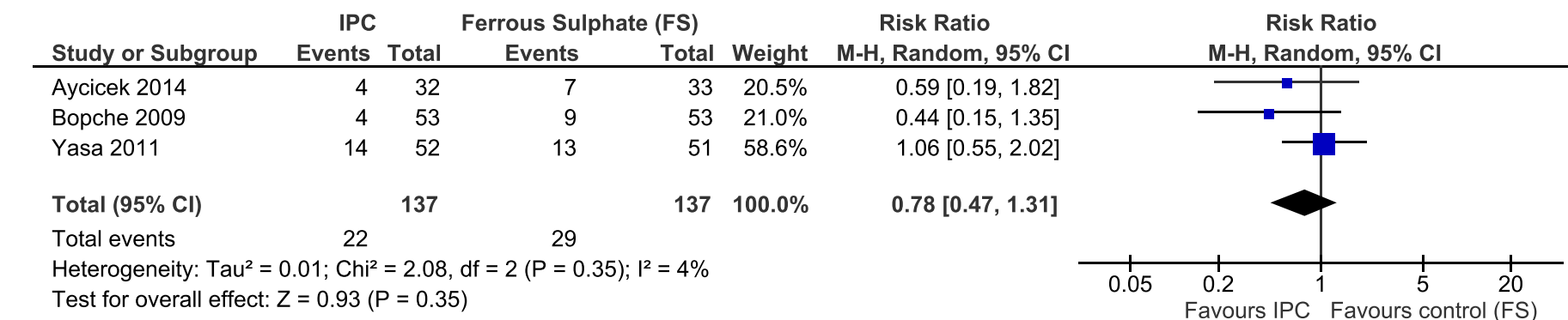

Supplement: Figure S1 — (A) Iron level. (B) MCV level. (C) MCH level. (D) Adverse effects (gastrointestinal disturbances). [file peerj-09-10527-s002.pdf]

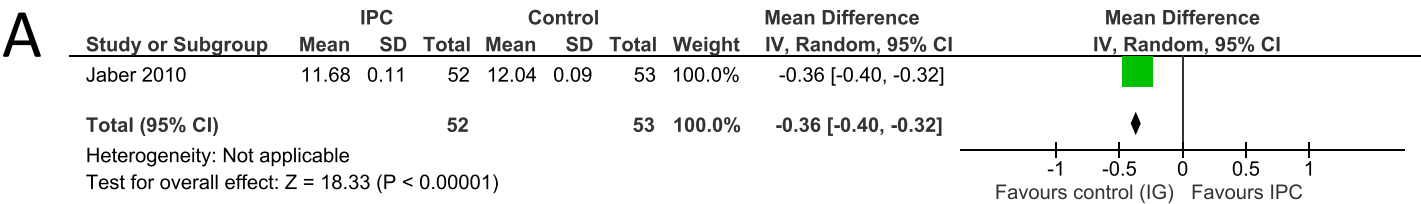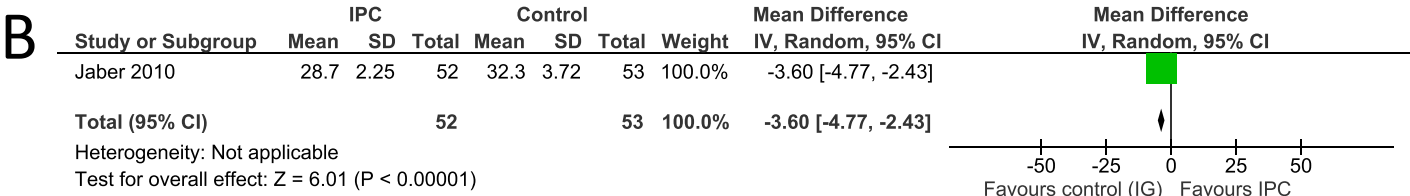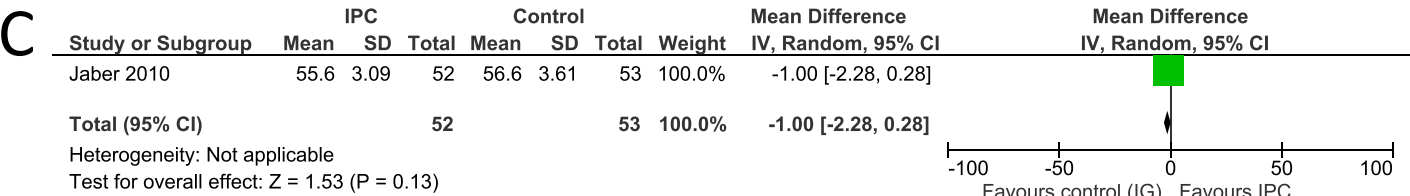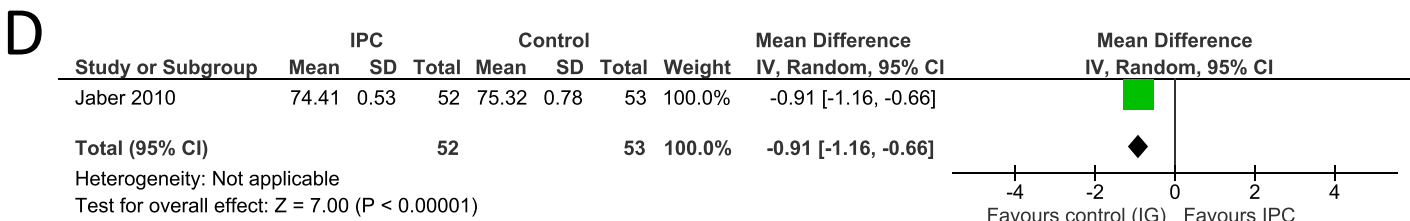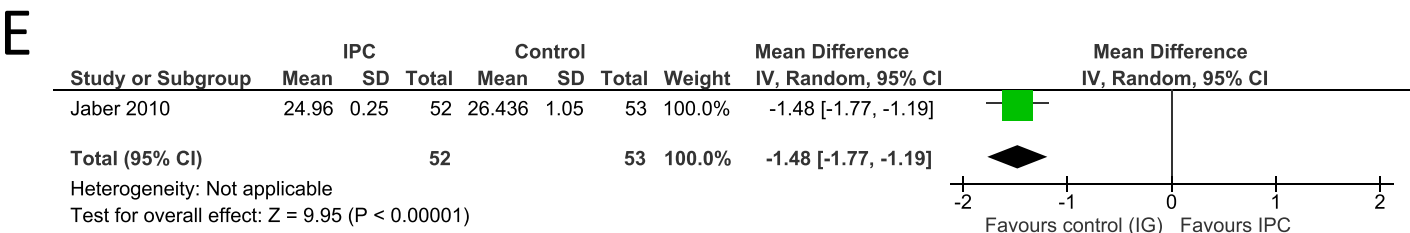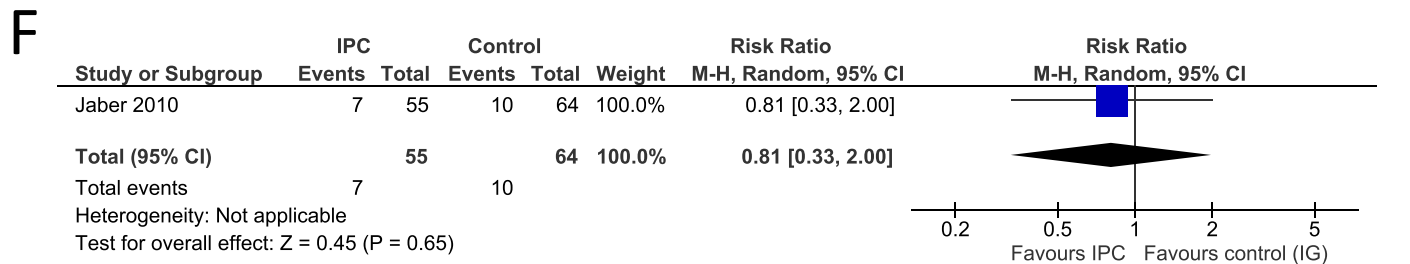

Supplement: Figure S2 — (A) Primary outcome: Hb level. (B) Primary outcome: Ferritin level. (C) Secondary outcome :Iron level. (D) Secondary outcome: MCV level. (E) Secondary outcome: MCH level. (F) Secondary outcome: Adverse effects. [file peerj-09-10527-s003.pdf]

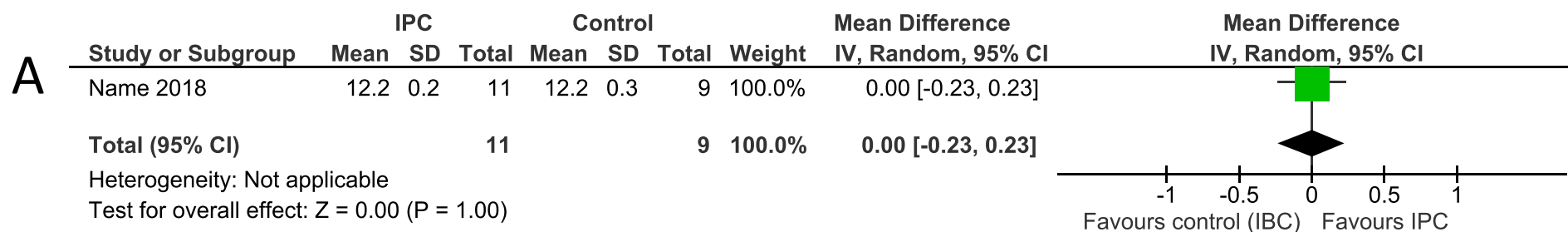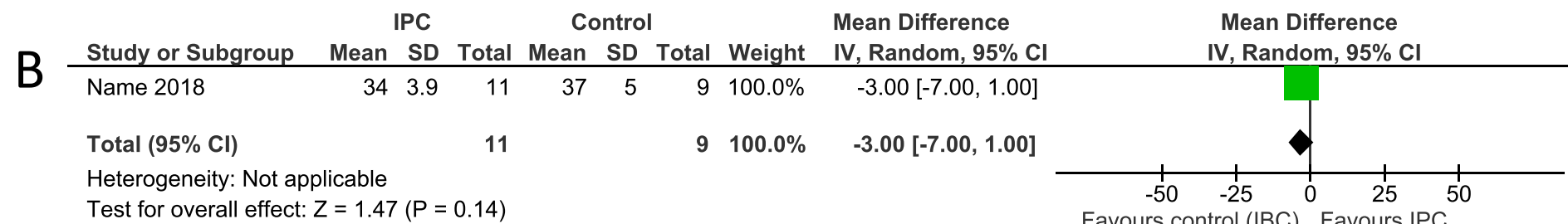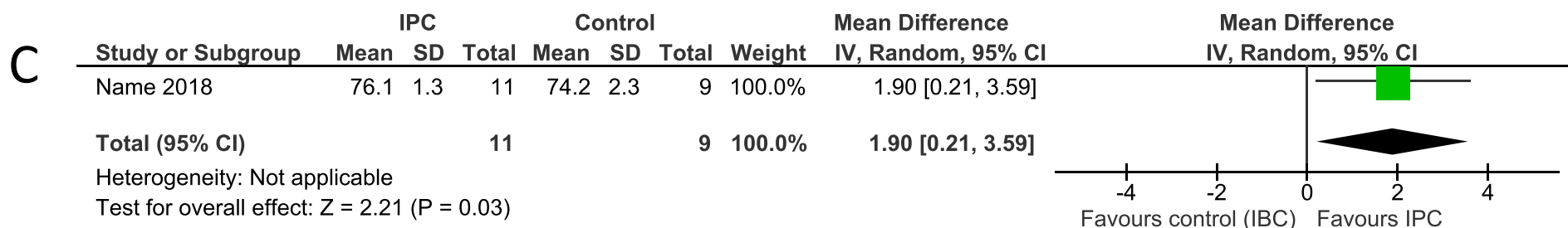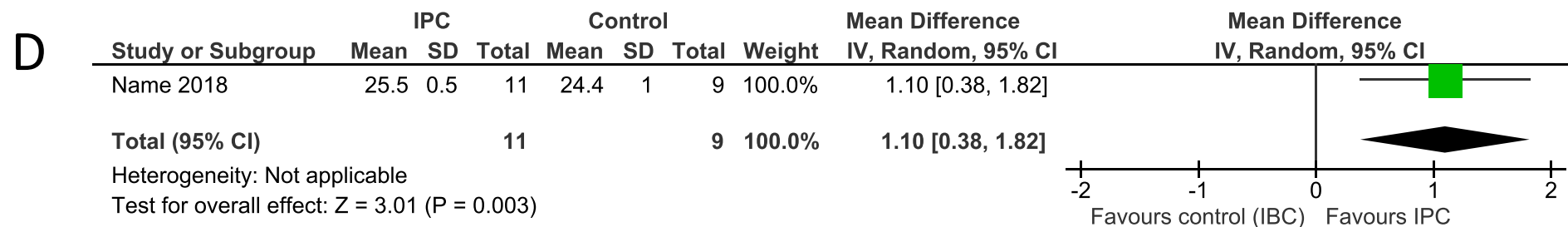

Supplement: Figure S3 — (A) Primary outcome: Hb level. (B) Primary outcome: Ferritin level. (C) Secondary outcome: MCV level. (D) Secondary outcome: MCH level. [file peerj-09-10527-s004.pdf]
