# Supplementary material for: Effectiveness of iron polymaltose complex in treatment and prevention of iron deficiency anemia in children: a systematic review and meta-analysis
Source: PeerJ. 2021 Jan 13;9:e10527. doi: 10.7717/peerj.10527 (PMC7811280; doi:10.7717/peerj.10527)
Supplement: Appendix S1 [file peerj-09-10527-s005.docx]

**APPENDIX 1. Search strategy**

**CENTRAL**

#1 (“iron deficiency anaemia”):ti,ab,kw

#2 (iron polymaltose complex OR ferrous III):ti,ab,kw

#3 (child* OR paed*):ti,ab,kw

#4 #1 AND #2 AND #3

**MEDLINE (PUBMED)**

1. "iron deficiency anaemia" AND “iron polymaltose” AND Paed* [All Fields]
2. "iron deficiency anaemia" AND “Ferr* III” AND Paed* [All Fields]
3. "iron deficiency anaemia" AND “iron polymaltose” AND Child*[All Fields]
4. "iron deficiency anaemia" AND “Ferr* III” AND Child*[All Fields]
