# Supplementary material for: Effectiveness of iron polymaltose complex in treatment and prevention of iron deficiency anemia in children: a systematic review and meta-analysis
Source: PeerJ. 2021 Jan 13;9:e10527. doi: 10.7717/peerj.10527 (PMC7811280; doi:10.7717/peerj.10527)
Supplement: Appendix S2 — PROSPERO registration number: CRD42019145020 [file peerj-09-10527-s006.pdf]

Iron polymaltose complex for iron deficiency anaemia in children

Ritzzaleena Rosli Mohd Rosli, Norhayati Mohd Noor

Citation

Ritzzaleena Rosli Mohd Rosli, Norhayati Mohd Noor. Iron polymaltose complex for iron deficiency anaemia in children. PROSPERO 2019 CRD42019145020 Available from: https://www.crd.york.ac.uk/prospero/display_record.php?ID=CRD42019145020

Review question

What is the effectiveness of oral iron polymaltose complex for treatment and prevention of children with iron deficiency anaemia in comparison with current standard treatment?

Searches

We will search the following sources from the inception of each database until recent.

1. Cochrane Central Register of Controlled Trials CENTRAL

2. Epistemonikos

3. MEDLINE

We will adapt the search strategy for other databases. We will only include English language papers or paper that has already been translated to English.

1. Iron deficiency anaemia

2. Iron polymaltose complex OR Ferrous III OR Ferric

3. Children OR Paedriatric

4. Treatment AND iron deficiency anaemia

5. Iron polymaltose complex AND iron deficiency anaemia

6. Iron deficiency anaemia AND iron polymaltose complex AND children OR paedriatric

Types of study to be included

Randomized control trials (RCTs) comparing oral iron polymaltose complex with standard oral iron supplement for treatment of iron deficiency anaemia. We will include quasi-RCTs, controlled clinical trials, blinded and open-label studies. Cross-over trials will be excluded.

Condition or domain being studied

Iron deficiency anaemia (IDA) is the most common nutritional deficiency affecting worldwide population in both developing and developed countries. Globally, anaemia affects 24.8% of the world population and has become among the major public health burden worldwide. Children in preschool-age has the highest prevalence, while men has the lowest prevalence with percentage of 47.4 and 12.7 respectively (WHO, 2008). In 2011, the prevalence of anaemia in children was 42.6% 95% (CI: 37—47), representing 273.2 million children (WHO, 2011a).

Participants/population

We will include children aged 0 to 17 (from birth until 17 years old) of either sex and of any ethnicity.

Intervention(s), exposure(s)

Supplementation of oral iron polymaltose complex. Oral IPC may be in solution/drops, syrup or tablet form.

We will consider any treatment or prophylaxis dose and regimen.

Comparator(s)/control
Any standard iron supplementations used for iron deficiency anaemia such as ferrous sulphate, iron gluconate and iron bisglycinate chelate.

Main outcome(s)
Primary outcomes

1. Haemoglobin level

2. Serum Ferritin level

* Measures of effect

For continuous data, we used the mean difference (MD) if outcomes were measured in the same way between trials. We used the standardised mean difference (SMD) to combine trials that measured the same outcome but used different methods; both with 95% confidence intervals (CIs).

Additional outcome(s)
Secondary outcomes

1. Serum iron level

2. Serum mean corpuscular volume (MCV)

3. Serum mean corpuscular haemoglobin (MCH)

4. Drugs adverse effects

* Measures of effect

For dichotomous data, we will measure the treatment effect and will present the results as summary risk ratio (RR) with 95% confidence intervals (CIs). For continuous data, we used the mean difference (MD) if outcomes were measured in the same way between trials. We used the standardised mean difference (SMD) to combine trials that measured the same outcome but used different methods; both with 95% confidence intervals (CIs).

Data extraction (selection and coding)
We designed a form to extract data. Two review authors (RRMR, NMN) will independently extract key participant and intervention characteristics. From each of the selected trials we will extract:

• study setting;

• participant characteristics (age, sex, ethnicity);

• methodology (number of participants randomised and analysed, duration of follow-up);

• method for diagnosing iron deficiency anaemia;

• Serum haemoglobin (Hb) level

• Serum ferritin level Serum iron level

• Mean corpuscular volume (MCV) level

• Mean corpuscular haemoglobin (MCH) level

• occurrence of related adverse events from treatment (e.g. gastrointestinal disturbances).

We will use Review Manager software (RevMan 2014) for data entry and checked for accuracy. Sources of trial funding and trialist declarations of interest will also be included.

Risk of bias (quality) assessment

Two review authors will independently assess risk of bias in all included studies using The Cochrane Collaboration's tool for assessing risk of bias outlined in the Cochrane Handbook for Systematic Reviews of Interventions (Higgins and Green, 2011) and contained in RevMan (RevMan 2014). Any disagreement will be resolved by discussion.

Strategy for data synthesis

We will carry out statistical analysis using the Review Manager software (RevMan 2014). We will use fixed?effect meta?analysis for combining data when good evidence shows homogeneous effects across trials of different methodological quality. Random?effects meta?analysis will be used if there is clinical heterogeneity sufficient to expect that the underlying treatment effects differ between trials, or if substantial statistical heterogeneity is detected. This will produce an overall summary, if an average treatment effect across trials is considered clinically meaningful.

The guide to interpretation of heterogeneity will be implemented as outlined (Higgins and Green, 2011).:

0% to 40% might not be important;

30% to 60% may represent moderate heterogeneity;

50% to 90% may represent substantial heterogeneity;

75% to 100% would be considerable heterogeneity.

For dichotomous data, we will measure the treatment effect and will present the results as summary risk ratio (RR) with 95% confidence intervals (CIs). For continuous data, we used the mean difference (MD) if outcomes were measured in the same way between trials. We used the standardised mean difference (SMD) to combine trials that measured the same outcome but used different methods; both with 95% confidence intervals (CIs).

Analysis of subgroups or subsets

We expect the following characteristics to introduce clinical heterogeneity, and we plan to conduct the following subgroup analyses if possible:

1. age of children, e.g. neonate, infant, toddler, adolescent

2. duration of treatment

3. dose of elemental iron

4. severity of iron deficiency anaemia at diagnosis

We will explore the potential sources of heterogeneity when important heterogeneity is present and either not pool the studies or use a random-effects model (DerSimonian and Laird, 1986)

Contact details for further information

Ritzzaleena Rosli Mohd Rosli
ritzz.rosli@student.usm.my

Organisational affiliation of the review

Hospital University Sains Malaysia

Review team members and their organisational affiliations

Dr Ritzzaleena Rosli Mohd Rosli. Hospital University Sains Malaysia

Assistant/Associate Professor Norhayati Mohd Noor. Hospital Universiti Sains Malaysia

Type and method of review
Meta-analysis, Systematic review

Anticipated or actual start date
14 July 2019

Anticipated completion date
14 June 2020

Funding sources/sponsors
None

Conflicts of interest

Language
English

Country
Malaysia

Stage of review
Review Ongoing

Subject index terms status
Subject indexing assigned by CRD

Subject index terms
Anemia, Iron-Deficiency; Child; Ferric Compounds; Humans

Date of registration in PROSPERO
29 October 2019

Date of first submission
28 July 2019

Stage of review at time of this submission

Stage	Started	Completed
Preliminary searches	No	No
Piloting of the study selection process	No	No
Formal screening of search results against eligibility criteria	Yes	No
Data extraction	No	No
Risk of bias (quality) assessment	No	No
Data analysis	No	No

The record owner confirms that the information they have supplied for this submission is accurate and complete and they understand that deliberate provision of inaccurate information or omission of data may be construed as scientific misconduct.

The record owner confirms that they will update the status of the review when it is completed and will add

publication details in due course.

Versions

29 October 2019

PROSPERO
This information has been provided by the named contact for this review. CRD has accepted this information in good faith and registered the review in PROSPERO. The registrant confirms that the information supplied for this submission is accurate and complete. CRD bears no responsibility or liability for the content of this registration record, any associated files or external websites.
