# Supplementary material for: Effectiveness of iron polymaltose complex in treatment and prevention of iron deficiency anemia in children: a systematic review and meta-analysis
Source: PeerJ. 2021 Jan 13;9:e10527. doi: 10.7717/peerj.10527 (PMC7811280; doi:10.7717/peerj.10527)
Supplement: Appendix S3 — A standardized form used to extract important data from the included trial. Randomized control trial 1: 2003 Sozmen. [file peerj-09-10527-s007.pdf]

Study Eligibility & Data Collection Form

General Information

**Study ID**<br>*(e.g. author name, year)*	Sozmen, 2003
**Form completed by**	Ritzzaleena Rosli Mohd Rosli
**Study author contact details**	ritzz.rosli@student.usm.my
**Publication type**<br>*(e.g. full report, abstract, letter)*	Full report
**List of included publications**	-
**References of similar trial***	-

*This is when the authors published the same study in several reports. All these references to a similar trial should be linked under one *Study ID* in RevMan.

Study eligibility

	Yes	No	Unclear	Further details
**RCT/Quasi/CCT**	/			RCT
**Relevant participants**	/			
**Relevant interventions**	/			
**Relevant outcomes***	/			

*Include only if the presence of outcomes form the inclusion criterion

If the above answers are 'YES', proceed to Section 1.

If any of the above answers are 'NO*', record below the information for 'Excluded studies'

Reason(s) for exclusion
-

Section 1. Characteristics of included studies

This section is to be completed by only one reviewer. State initials: RRMR

**METHODS**	**Descriptions as stated in paper**
**Aim of study** *(e.g. efficacy, equivalence, pragmatic)*	The aim of this study was to investigate the effects of ferrous sulphate and ferric hydroxide–polymaltose complex supplementation on the trace element status in children with IDA.
**Design** *(e.g. parallel, crossover, cluster)*	Parallel, randomised group study of Iron Polymaltose versus Ferrous sulphate
**Unit of allocation** *(by individuals, cluster/ groups or body parts)*	individuals
**Start & end dates**	Not mentioned
**Total study duration**	Total study duration not stated. Treatment duration was 6 months
**Sources of funding** *(including role of funders)*	-
**Possible conflicts of interest** *(for study authors)*	-

**PARTICIPANTS**	**Description** *(include information for each intervention or comparison group)*
**Population description** *(Company/companies; occupation)*	Children with iron deficiency anaemia
**Setting** *(including location (city, state, country) and single centre / multicenter)*	Turkey
**Inclusion criteria**	1. Aged 8-168 months<br>2. Diagnosed with IDA by a haematologist according to their clinical and laboratory data: "*Hb<11.5 g/dL, Htc <35%, MCV<75, ferritin<20 mg/dL*"
**Exclusion criteria**	-
**Method of recruitment of participants** *(e.g. phone, mail, clinic patients, voluntary)*	-
**Total no. randomised**	37
**Clusters** *(if applicable, no., type, no. people per cluster)*	-

**No. randomised per group** *(specify whether no. people or clusters)*	Intervention: 17<br>Control: 20
**No. missing** *(if overall, e.g. exclusions & withdrawals, whether or not missing from analysis)*	Intervention: 3<br>Control: 9
**Reasons missing**	Not mentioned
**Baseline imbalances**	No baseline imbalances of the laboratory's values.
**Age**	8- 168 months
**Sex (proportion)**	Male 22 female 15
**Race/Ethnicity**	-
**Other relevant sociodemographics**	-
**Subgroups measured** *(eg split by age or sex)*	-
**Subgroups reported**	-

Section 2. Risk of bias assessment

We recommend you refer to and use the method described in the Cochrane Handbook.

This section is completed by two reviewers. State initials: (i) RRMR (ii) NMN

**Domain**	**Risk of bias**	**Support for judgement** *(include direct quotes where available with explanatory comments)*	**Location in text or source** *(page, table)*
**Random sequence generation** *(selection bias)*	unclear	*"Seventeen, who were chosen randomly,"*	Page 3
**Allocation concealment** *(selection bias)*	unclear	Not mentioned in full text	-
**Blinding of participants and personnel** *(performance bias)*	unclear	Not mentioned in full text	-
**Blinding of outcome assessment** *(detection bias)*	low	*"Hemoglobin and hematocrit levels were determined by an automatic hemocounter (Abbott Cell-Dyne 3700 hemocounter), and serum iron and iron-binding capacity were measured by an autoanalyzer using a commercially available kit."*	Page 3
**Incomplete outcome data** *(attrition bias)*	unclear	Reason for missing not mentioned in full text.<br>However, total final number of participants in both groups is balanced.<br>*"Thirty-seven children (aged 8–168 mo, 22 boys and 15 girls) with IDA were taken into the study"*<br>*"Seventeen, who were chosen randomly, were treated with the ferric hydroxide-polymaltose complex (Ferrum, Vifor, Switzerland) (6 mg/kg/d in the first 3 mo for initial therapy and then 3 mg/kg for 3 mo as maintenance); the others (n=20) were treated with a ferrous sulfate complex"*	Page 2<br>Page 3
**Selective outcome reporting** *(reporting bias)*	Low	Study protocol not available.<br>All pre-specified and expected outcomes of interest are reported	-
**Other bias**	Low	No other bias identified	-

Random sequence generation = Process used to assign people into intervention and control groups
Allocation concealment = Process used to prevent foreknowledge of group assignment in a RCT
Blinding of participants and personnel = Presence or absence of blinding for participants and health personnel
Blinding of outcome assessment = presence or absence of blinding for assessment of outcome
Incomplete outcome data = application of intention-to-treat analysis is one in which all the participants in a trial are analysed according to the intervention to which they were allocated
Selective outcome reporting = Selection of a subset of the original variables recorded

Section 3. Intervention groups

This section is completed by two reviewers. State initials: (i) RRMR (ii) NMN

**Outcomes relevant to your review** *(Copy and paste from 'Types of outcome measures')*	**Reported in paper** *(Yes / No)*	**Outcome definition** *(with diagnostic criteria if relevant)*	**Unit of measurement & tool** *(if relevant)*	**Reanalysis required?** *(specify)*
1. Haemoglobin (Hb)	Yes	level at end of treatment	(g/dL) automatic hemocounter (Abbott-Cell-Dyne 3700 hemocounter)	No
2. Serum Ferritin	Yes	level at end of treatment	Not written in table	No
3. Serum iron	Yes	level at end of treatment	Not written in table	No
4. Serum mean corpuscular volume (MCV)	No	-	-	No
5. Serum mean corpuscular haemoglobin (MCH)	No	-	-	No
6. Gastrointestinal disturbances as side effects	No	-	-	No

Section 4. Data and analysis

**DICHOTOMOUS OUTCOME**	Intervention group		Control group	
	Number of events	Number of participants	Number of events	Number of participants
-	-	-	-	-

State details if outcomes were only described in text or figures.

**CONTINUOUS OUTCOME**	Unit of measurement	Intervention group		Control group	
		n	Mean (SD)	n	Mean (SD)
1. Haemoglobin (Hb)	(g/dL)	14	11.6 (0.7)	11	12.5(1.2)
2. Serum Ferritin	ng/mL	14	11.8 (7.8)	11	56.6 (43.1)
3. Serum iron	ng/dL	14	48.9 (39.8)	11	103.1 (86.3)

State details if outcomes were only described in text or figures.

Section 5. Other information

	Description as stated in paper
**Key conclusions of study authors**	*"In conclusion, our data showed that copper and ceruloplasmin metabolisms were affected by ferrous iron supplementation, possibly by a competition, whereas ferric iron maintained normal levels of zinc. We conclude that copper and zinc status of patients with IDA should be taken into consideration before and after iron therapy."*
**Results that you calculated using a formula**	-
**References to other relevant studies** *(Did this report include any references to unpublished data from potentially eligible trials not already identified for this review? If yes, give list contact name and details)*	-
**Correspondence required for further study information** *(from whom, what and when)*	-

Sources:

Higgins JPT, Green S (editors). Cochrane Handbook for Systematic Reviews of Interventions Version 5.1.0 [updated March 2011]. The Cochrane Collaboration, 2011.Available from www.cochrane-handbook.org.
