# Supplementary material for: Effectiveness of iron polymaltose complex in treatment and prevention of iron deficiency anemia in children: a systematic review and meta-analysis
Source: PeerJ. 2021 Jan 13;9:e10527. doi: 10.7717/peerj.10527 (PMC7811280; doi:10.7717/peerj.10527)
Supplement: Appendix S4 — A standardized form used to extract important data from the included trial. Randomized control trial 2: 2004 Kavakli. [file peerj-09-10527-s008.pdf]

Study Eligibility & Data Collection Form

General Information

**Study ID**<br>*(e.g. author name, year)*	Kavakli 2004
**Form completed by**	Ritzzaleena Rosli Mohd Rosli
**Study author contact details**	ritzz.rosli@student.usm.my
**Publication type**<br>*(e.g. full report, abstract, letter)*	Full report
**List of included publications**	-
**References of similar trial***	-

*This is when the authors published the same study in several reports. All these references to a similar trial should be linked under one *Study ID* in RevMan.

Study eligibility

	Yes	No	Unclear	Further details
**RCT/Quasi/CCT**	/			RCT
**Relevant participants**	/			
**Relevant interventions**	/			
**Relevant outcomes***	/			

*Include only if the presence of outcomes form the inclusion criterion

If the above answers are 'YES', proceed to Section 1.

If any of the above answers are 'NO*', record below the information for 'Excluded studies'

Reason(s) for exclusion
-

Section 1. Characteristics of included studies

This section is to be completed by only one reviewer. State initials: RRMR

**METHODS**	**Descriptions as stated in paper**
**Aim of study** *(e.g. efficacy, equivalence, pragmatic)*	The first aim of this study is to investigate the oxidant effects of oral iron supplementation through determining the LDL oxidation and copper stimulated LDL oxidation as well as antioxidant status and MDA levels in erythrocytes of children with IDA.<br>The second aim of this study is to determine the possible side effects of different iron preparations during the treatment process.
**Design** *(e.g. parallel, crossover, cluster)*	Parallel, randomised group study of Fe2+ (Ferro-Sanol suspension) and Fe3+ (Ferrum suspension)
**Unit of allocation** *(by individuals, cluster/ groups or body parts)*	Individuals
**Start & end dates**	November 1999 and March 2001
**Total study duration**	Total study duration was 1 year 3 months. Treatment duration was 6 months
**Sources of funding** *(including role of funders)*	-
**Possible conflicts of interest** *(for study authors)*	-

**PARTICIPANTS**	**Description** *(include information for each intervention or comparison group)*
**Population description** *(Company/companies; occupation)*	Children with iron deficiency anaemia
**Setting** *(including location (city, state, country) and single centre / multicenter)*	Pediatrics Outpatient<br>Clinic in Ege University, Turkey.
**Inclusion criteria**	1. The children with IDA, aged between 6 months and 15 years<br>2. IDA was defined as hemoglobin (Hb) below 10 g/dL for children below or at the age of 12 months and below 11 g/dL for children older than 12 months age, haematocrit below 35%, transferrin saturation below 16%, and a serum ferritin value below 20 ng/mL.

**Exclusion criteria**	1. iron preparations in last 3 months<br>2. had acute infection<br>3. had a history of chronic disease, parasites, suffered blood loss for any reason<br>4. had occult blood in stools
**Method of recruitment of participants** *(e.g. phone, mail, clinic patients, voluntary)*	Consecutive patients who attended the Pediatrics Outpatient Clinic in Ege University, Turkey
**Total no. randomised**	72
**Clusters** *(if applicable, no., type, no. people per cluster)*	-
**No. randomised per group** *(specify whether no. people or clusters)*	Intervention: 33 patients (male/female: 26/7).<br>Control: 39 patients (male/female: 23/16)
**No. missing** *(if overall, e.g. exclusions & withdrawals, whether or not missing from analysis)*	Intervention: 14<br>Control: 19
**Reasons missing**	Did not continue the treatment after 3rd month.
**Baseline imbalances**	There was no difference between the patients who completed 6th month treatment and those who did not, according to their age, sex, and initial, 1st, and 3rd month parameters evaluated.
**Age**	8- 168 months
**Sex (proportion)**	Intervention: 19; male/female:16/3<br>Control: 20; male/female: 9/11
**Race/Ethnicity**	-
**Other relevant sociodemographics**	-
**Subgroups measured** *(eg split by age or sex)*	-
**Subgroups reported**	-

Section 2. Risk of bias assessment
We recommend you refer to and use the method described in the Cochrane Handbook.

This section is completed by two reviewers. State initials: (i) RRMR (ii) NMN

**Domain**	**Risk of bias**<br><br>High<br>Unclear<br>Low	**Support for judgement**<br>*(include direct quotes where available with explanatory comments)*	**Location in text or source**<br>*(page, table)*
**Random sequence generation**<br>*(selection bias)*	unclear	*"were randomly included in Fe2+ and Fe3+group,"*<br>Comment: Not clearly describe.	Page 2
**Allocation concealment**<br>*(selection bias)*	unclear	Not mentioned in full text	-
**Blinding of participants and personnel**<br>*(performance bias)*	unclear	Not mentioned in full text	-
**Blinding of outcome assessment**<br>*(detection bias)*	low	*"Hemogram and ferritin levels were detected on the same day."*	Page 3
**Incomplete outcome data**<br>*(attrition bias)*	low	"*While 20 of the children in the Fe2+ group (male/female: 9/11) and Fe3+ 19 of the children in the group (male/female:16/3) completed the whole duration of the study, 19 patients in the Fe2+ group and 14 in the Fe3+ group did not continue the treatment after 3rd month."*<br><br>*"There was no difference between the patients who completed 6th month treatment and those who did not, according to their age, sex, and initial, 1st, and 3rd month parameters evaluated."*	Page 4
**Selective outcome reporting**<br>*(reporting bias)*	low	Study protocol not available. However, all pre-specified and expected outcomes of interest are reported	-

**Other bias**	low	No other bias identified.	-

Random sequence generation = Process used to assign people into intervention and control groups
Allocation concealment = Process used to prevent foreknowledge of group assignment in a RCT
Blinding of participants and personnel = Presence or absence of blinding for participants and health personnel
Blinding of outcome assessment = presence or absence of blinding for assessment of outcome
Incomplete outcome data = application of intention-to-treat analysis is one in which all the participants in a trial are analysed according to the intervention to which they were allocated
Selective outcome reporting = Selection of a subset of the original variables recorded

Section 3. Intervention groups

This section is completed by two reviewers. State initials: (i) RRMR (ii) NMN

**Outcomes relevant to your review** *(Copy and paste from 'Types of outcome measures')*	**Reported in paper** *(Yes / No)*	**Outcome definition** *(with diagnostic criteria if relevant)*	**Unit of measurement & tool** *(if relevant)*	**Reanalysis required?** *(specify)*
1. Hemoglobin (Hb)	Yes	level at end of treatment	(g/dL)	No
2. Serum Ferritin	No	-	-	-
3. Serum iron	No	-	-	-
4. Serum mean corpuscular volume (**MCV**)	No	-	-	-
5. Serum mean corpuscular hemoglobin (**MCH**)	No	-	-	-
6. Gastrointestinal disturbances as side effects	Yes	stomachache, constipation, diarrhea, and nausea	-	No

Section 4. Data and analysis

DICHOTOMOUS OUTCOME	Intervention group		Control group	
	Number of events	Number of participants	Number of events	Number of participants
Gastrointestinal disturbances as side effects	8	19	5	20

State details if outcomes were only described in text or figures.

CONTINUOUS OUTCOME	Unit of measurement	Intervention group		Control group	
		n	Mean (SD)	n	Mean (SD)
1. Hemoglobin (Hb)	(g/dL)	19	11.4 (1.2)	20	12.5(1.2)

State details if outcomes were only described in text or figures.

Section 5. Other information

	**Description as stated in paper**
**Key conclusions of study authors**	When the Fe2+ type of iron is used in children, no toxic effect arises in terms of the increase in free oxygen radicals. Therefore, the first choice for the treatment of IDA in childhood must be Fe2+ sulfate preparations, because they are more efficient, do not cause any toxic effects, and are considerably cheaper products.
**Results that you calculated using a formula**	-
**References to other relevant studies** *(Did this report include any references to unpublished data from potentially eligible trials not already identified for this review? If yes, give list contact name and details)*	-
**Correspondence required for further study information** *(from whom, what and when)*	-

Sources:

Higgins JPT, Green S (editors). Cochrane Handbook for Systematic Reviews of Interventions Version 5.1.0 [updated March 2011]. The Cochrane Collaboration, 2011.Available from www.cochrane-handbook.org.
