# Supplementary material for: Effectiveness of iron polymaltose complex in treatment and prevention of iron deficiency anemia in children: a systematic review and meta-analysis
Source: PeerJ. 2021 Jan 13;9:e10527. doi: 10.7717/peerj.10527 (PMC7811280; doi:10.7717/peerj.10527)
Supplement: Appendix S5 — A standardized form used to extract important data from the included trial. Randomized control trial 3: 2009 Bopche. [file peerj-09-10527-s009.pdf]

Study Eligibility & Data Collection Form

General Information

**Study ID**<br>*(e.g. author name, year)*	Bopche, 2009
**Form completed by**	Ritzzaleena Rosli Mohd Rosli
**Study author contact details**	ritzz.rosli@student.usm.my
**Publication type**<br>*(e.g. full report, abstract, letter)*	Full report
**List of included publications**	-
**References of similar trial***	-

*This is when the authors published the same study in several reports. All these references to a similar trial should be linked under one *Study ID* in RevMan.

Study eligibility

	Yes	No	Unclear	Further details
**RCT/Quasi/CCT**	/			RCT
**Relevant participants**	/			
**Relevant interventions**	/			
**Relevant outcomes***	/			

*Include only if the presence of outcomes form the inclusion criterion

If the above answers are 'YES', proceed to Section 1.

If any of the above answers are 'NO*', record below the information for 'Excluded studies'

Reason(s) for exclusion
-

Section 1. Characteristics of included studies

This section is to be completed by only one reviewer. State initials: RRMR

METHODS	Descriptions as stated in paper
**Aim of study** *(e.g. efficacy, equivalence, pragmatic)*	To determines the therapeutic efficacy of two different oral iron preparations: iron polymaltose complex (IPC) and ferrous sulfate (FS).
**Design** *(e.g. parallel, crossover, cluster)*	Parallel study comparing Iron Polymaltose and Ferrous Sulphate
**Unit of allocation** *(by individuals, cluster/ groups or body parts)*	individuals
**Start & end dates**	October 2004 – September 2005
**Total study duration**	1 year
**Sources of funding** *(including role of funders)*	None
**Possible conflicts of interest** *(for study authors)*	Not stated

PARTICIPANTS	**Description** *(include information for each intervention or comparison group)*
**Population description** *(Company/companies; occupation)*	Children with Iron deficiency anaemia
**Setting** *(including location (city, state, country) and single centre / multicenter)*	Teaching institution with a tertiary level paediatric centre in central India.
**Inclusion criteria**	1. Age ranging from 1-6 years<br>2. IDA confirmed by serum iron chemistry
**Exclusion criteria**	Not mentioned
**Method of recruitment of participants** *(e.g. phone, mail, clinic patients, voluntary)*	Patients attending outpatient department
**Total no. randomised**	118
**Clusters** *(if applicable, no., type, no. people per cluster)*	No

**No. randomised per group** *(specify whether no. people or clusters)*	Intervention: 59<br>Control: 59
**No. missing** *(if overall, e.g. exclusions & withdrawals, whether or not missing from analysis)*	Intervention: 6<br>Control:6
**Reasons missing**	Lost to follow up
**Baseline imbalances**	No
**Age**	1-6 years old
**Sex (proportion)**	Not mentioned
**Race/Ethnicity**	Indian
**Other relevant sociodemographic**	-
**Subgroups measured** *(e.g. split by age or sex)*	-
**Subgroups reported**	-

Section 2. Risk of bias assessment

We recommend you refer to and use the method described in the Cochrane Handbook.

This section is completed by two reviewers. State initials: (i) RRMR (ii) NMN

**Domain**	**Risk of bias**			**Support for judgement** *(include direct quotes where available with explanatory comments)*	**Location in text or source** *(page, table)*
	Low	High	Unclear		
**Random sequence generation** *(selection bias)*	Low			*"Randomization was achieved by simple randomization"*	Page 2
**Allocation concealment** *(selection bias)*	Low			*"and allocation was concealed by sealed envelope technique."*	Page 2
**Blinding of participants and personnel** *(performance bias)*	Unclear			Not mentioned in full text	-
**Blinding of outcome assessment** *(detection bias)*	Low			Outcome is base on laboratory results from blood sample.<br><br>Comment: Objective / continuous outcome	Page 2
**Incomplete outcome data** *(attrition bias)*	Low			Number of missing participants are equal in each group and both for similar reason.	Figure 1: study flow chart, page 2
**Selective outcome reporting** *(reporting bias)*	Low			Study protocol not available<br><br>All pre-specified and expected outcomes of interest are reported	-
**Other bias**	Low			No other bias identified	-

Random sequence generation = Process used to assign people into intervention and control groups
Allocation concealment = Process used to prevent foreknowledge of group assignment in a RCT
Blinding of participants and personnel = Presence or absence of blinding for participants and health personnel
Blinding of outcome assessment = presence or absence of blinding for assessment of outcome
Incomplete outcome data = application of intention-to-treat analysis is one in which all the participants in a trial are analysed according to the intervention to which they were allocated
Selective outcome reporting = Selection of a subset of the original variables recorded

Section 3. Intervention groups

This section is completed by two reviewers. State initials: (i) RRMR (ii) NMN

**Outcomes relevant to your review** *(Copy and paste from 'Types of outcome measures')*	**Reported in paper** *(Yes / No)*	**Outcome definition** *(with diagnostic criteria if relevant)*	**Unit of measurement & tool** *(if relevant)*	**Reanalysis required?** *(specify)*
1. Haemoglobin (Hb)	Yes	level at end of treatment	(g/dL)	No
2. Serum Ferritin	No	-	-	No
3. Serum iron	No	-	-	No
4. Serum mean corpuscular volume (MCV)	No	-	-	No
5. Serum mean corpuscular haemoglobin (MCH)	No	-	-	No
6. Gastrointestinal disturbances as side effects	Yes	Described as gastrointestinal side effects.	-	No

Section 4. Data and analysis

**DICHOTOMOUS OUTCOME**	Intervention group		Control group	
	Number of events	Number of participants	Number of events	Number of participants
Gastrointestinal disturbances as side effects	4	53	9	53

State details if outcomes were only described in text or figures.

**CONTINUOUS OUTCOME**	Unit of measurement	Intervention group		Control group	
		n	Mean (SD)	n	Mean (SD)
Mean change of haemoglobin (Hb)	(g/dL)	53	8.565 (0.3809)	53	8.985 (1.6507)
Haemoglobin (Hb)	(g/dL)	53	8.67 (0.73)	53	9.44 (0.67)

State details if outcomes were only described in text or figures.

Section 5. Other information

	**Description as stated in paper**
**Key conclusions of study authors**	Ferrous sulfate has a better clinical response as compared to Iron polymaltose complex for treating iron deficiency anemia in children.
**Results that you calculated using a formula**	-
**References to other relevant studies** *(Did this report include any references to unpublished data from potentially eligible trials not already identified for this review? If yes, give list contact name and details)*	-
**Correspondence required for further study information** *(from whom, what and when)*	-

Sources:

Higgins JPT, Green S (editors). Cochrane Handbook for Systematic Reviews of Interventions Version 5.1.0 [updated March 2011]. The Cochrane Collaboration, 2011.Available from www.cochrane-handbook.org.
