# Supplementary material for: Effectiveness of iron polymaltose complex in treatment and prevention of iron deficiency anemia in children: a systematic review and meta-analysis
Source: PeerJ. 2021 Jan 13;9:e10527. doi: 10.7717/peerj.10527 (PMC7811280; doi:10.7717/peerj.10527)
Supplement: Appendix S6 — A standardized form used to extract important data from the included trial. Randomized control trial 4: 2010 Jaber. [file peerj-09-10527-s010.pdf]

Study Eligibility & Data Collection Form

General Information

**Study ID**<br>*(e.g. author name, year)*	Jaber, 2010
**Form completed by**	Ritzzaleena Rosli Mohd Rosli
**Study author contact details**	ritzz.rosli@student.usm.my
**Publication type**<br>*(e.g. full report, abstract, letter)*	Full report
**List of included publications**	-
**References of similar trial***	-

*This is when the authors published the same study in several reports. All these references to a similar trial should be linked under one *Study ID* in RevMan.

Study eligibility

	Yes	No	Unclear	Further details
**RCT/Quasi/CCT**	/			RCT
**Relevant participants**	/			
**Relevant interventions**	/			
**Relevant outcomes***	/			

*Include only if the presence of outcomes form the inclusion criterion

If the above answers are 'YES', proceed to Section 1.

If any of the above answers are 'NO*', record below the information for 'Excluded studies'

Reason(s) for exclusion
-

Section 1. Characteristics of included studies

This section is to be completed by only one reviewer. State initials: RRMR

METHODS	Descriptions as stated in paper
**Aim of study** *(e.g. efficacy, equivalence, pragmatic)*	To evaluates the effectiveness of Iron polymaltose complex (IPC) and iron gluconate (IG) preparation in the prevention of ferropenic anaemia in healthy infants
**Design** *(e.g. parallel, crossover, cluster)*	Parallel, randomised group study of Iron Polymaltose versus Iron Gluconate
**Unit of allocation** *(by individuals, cluster/ groups or body parts)*	Individuals
**Start & end dates**	March 2007 until January 2009
**Total study duration**	22 months
**Sources of funding** *(including role of funders)*	Not mentioned
**Possible conflicts of interest** *(for study authors)*	

PARTICIPANTS	Description<br>*(include information for each intervention or comparison group)*
**Population description** *(Company/companies; occupation)*	healthy infants attending a community paediatric centre
**Setting** *(including location (city, state, country) and single center / multicenter)*	Community pediatric center in Israel (single center)
**Inclusion criteria**	(1). All were term infants;<br>(2). Birth weight >2500 g;<br>(3). No major congenital abnormalities, or systemic diseases;<br>(4). Naive to iron supplement treatment;<br>(5) Agreement by parent/guardian to refrain from treating the infant with any other mineral or vitamin supplement except vitamins A and /or D for the duration of the study;<br>(6) Reasonable parent/guardian level of knowledge of Hebrew, English, or Arabic (speak, read, write);<br>(7) Acknowledgment by the parent or guardian that they understand the study procedures and agree to complete daily records on tolerance and stool characteristics during the study period;<br>(8) Receipt by the study researchers of a voluntarily signed informed consent form from the parent/guardian.

**Exclusion criteria**	1. Infants receiving any medication (other than topical ointments, or vitamins A and/or D)<br>2. Infants who had a strong family history of allergies or chronic disease (e.g., congenital heart disease, diabetes),<br>3. Carriers of the thalassemia gene<br>4. participants participating in any other interventional clinical study<br>5. Failed to keep their next appointment.
**Method of recruitment of participants** *(e.g. phone, mail, clinic patients, voluntary)*	Healthy infants attending a community pediatric center in Israel
**Total no. randomized**	123
**Clusters** *(if applicable, no., type, no. people per cluster)*	-
**No. randomized per group** *(specify whether no. people or clusters)*	Intervention: 57<br>Control: 66
**No. missing** *(if overall, e.g. exclusions & withdrawals, whether or not missing from analysis)*	Intervention:5<br>Control: 13
**Reasons missing**	Intervention: 2 thalassemia carrier, 3 dropped out<br>Control: 2 thalassemia carrier, 11 dropped out
**Baseline imbalances**	There were no significant differences between the IG and IPC groups in anthropometric characteristics or socioeconomic status. The laboratory values are shown no differences between the 2 groups were noted in serum ferritin, serum transferrin, hematocrit, MCV, MCH, or RDW.
**Age**	120- 180 days (inclusive)
**Sex (proportion)**	Intervention: Male 30; female 22<br>Control: Male 30; Female 23
**Race/Ethnicity**	Not mentioned

**Other relevant sociodemographic**	Mother's education, [n (%)]<br>> 8 y: Intervention 3 (6.7) Control 3 (6.7)<br>8-12 y Intervention 19 (42.0) Control 10 (22.7)<br>College or university: Intervention 23 (51.0) Control 31 (70.5)<br>Father's education, [n (%)]<br>>8 y: Intervention 2 (4.5) Control 2 (4.5)<br>8-12 y: Intervention 31 (70.5) Control 20 (45.5) 0.04<br>College or university: Intervention 11 (25) Control 22 (50.3)<br>Mother's occupation, [n (%)]<br>Unemployed: Intervention 28 (60.9) Control 27 (64.3)<br>Salaried worker: Intervention 18 (39) Control 14 (33.3) 0.5<br>Self-employed or academic: Intervention 0 Control 1 (2.4)<br>Father's occupation, [n (%)]<br>Unskilled worker: Intervention 9 (21.9) Control 9 (21.9)<br>Unemployed 2: Intervention (4.8) 2 Control (4.8) 1.1<br>Salaried worker: Intervention 10 (24.4) Control 10 (24.4)<br>Self-employed or academic 20 (48.8) Control 20 (48.8)<br>Mother's age (y),<br>mean +/- SEM Intervention 26.7+/-0.8 Control 27.3+/-0.89<br>Father's age (y),<br>mean +/- SEM Intervention 32.2+/-0.93 Control 32.3+/-0.79 0.01
**Subgroups measured** *(e.g. split by age or sex)*	-
**Subgroups reported**	-

Section 2. Risk of bias assessment

We recommend you refer to and use the method described in the Cochrane Handbook.

This section is completed by two reviewers. State initials: (i) RRMR (ii) NMN

**Domain**	**Risk of bias**<br>Low<br>High<br>Unclear	**Support for judgement** *(include direct quotes where available with explanatory comments)*	**Location in text or source** *(page, table)*
**Random sequence generation** *(selection bias)*	unclear	*"The infant was randomly allocated to preventive treatment by 1 of 2 groups of pediatricians"*	Page 2
**Allocation concealment** *(selection bias)*	unclear	*"Participants assigned to pediatricians 01 were prescribed IPC (Ferripel-3; CTS Chemical Ind. Ltd., Israel). Ferripel-3 is an iron preparation that contains nonionic ferric iron (III) and hydroxide polymaltose in a stable complex. Those assigned to pediatricians 02 were prescribed IG (Ferro 15; Sam-On Ltd., Israel)."*<br><br>Comments: not clearly explained.	Page 2
**Blinding of participants and personnel** *(performance bias)*	unclear	Not mentioned in full text	-

**Blinding of outcome assessment** *(detection bias)*	low	"*Blood samples were collected for measurement of serum levels of iron ferritin and transferrin and MCV, mean corpuscular hemoglobin (MCH), hematocrit, and red cell distribution width (RDW).*"	Page 2
**Incomplete outcome data** *(attrition bias)*	low	The final study groups comprised of balanced number of participants.<br><br>*"Of the 123 infants initially recruited for the study, 66 were treated with IG and 57 with IPC. During the course of the study, 4 patients were found to be carriers of thalassemia (2 in each group) and were excluded from the laboratory analysis. In addition, 14 infants dropped out (11 in the IG group, 3 in the IPC group). The final study groups comprised 53 infants in the IG group and 52 in the IPC group."*	Page 3
**Selective outcome reporting** *(reporting bias)*	low	Study protocol not available.<br>In methods:<br>*1. "iron ferritin and transferrin and MCV, mean corpuscular hemoglobin (MCH), hematocrit, and red cell distribution width (RDW)."*<br>*2. "the parent/guardian was given a daily diary and asked to document throughout the study period any symptoms of gastrointestinal dysfunction in the infant, such as flatulence, vomiting, spitting, tooth staining, constipation, and diarrhea."*<br><br>In results:<br>1. Hb, iron, ferritin, transferrin, MCV, MCH, RDW, Hct, transferrin.<br>2. Spitting, vomiting, constipation.	Page 2<br><br>Page 3, Table 2<br><br>Page 3, table 3.
**Other bias**	low	No other bias identified	

Random sequence generation = Process used to assign people into intervention and control groups
Allocation concealment = Process used to prevent foreknowledge of group assignment in a RCT
Blinding of participants and personnel = Presence or absence of blinding for participants and health personnel
Blinding of outcome assessment = presence or absence of blinding for assessment of outcome
Incomplete outcome data = application of intention-to-treat analysis is one in which all the participants in a trial are analyzed according to the intervention to which they were allocated
Selective outcome reporting = Selection of a subset of the original variables recorded

Section 3. Intervention groups

This section is completed by two reviewers. State initials: (i) RRMR (ii) NMN

**Outcomes relevant to your review** *(Copy and paste from 'Types of outcome measures')*	**Reported in paper** *(Yes / No)*	**Outcome definition** *(with diagnostic criteria if relevant)*	**Unit of measurement & tool** *(if relevant)*	**Reanalysis required?** *(specify)*
1. Hemoglobin (Hb)	Yes	level at end of treatment	(g/dL)	No
2. Serum Ferritin	Yes	level at end of treatment	ng/mL	No
3. Serum iron	Yes	level at end of treatment	ng/dL	No
4. Serum mean corpuscular volume (**MCV**)	Yes	level at end of treatment. Mean +/- SEM	fL	No
5. Serum mean corpuscular hemoglobin (**MCH**)	Yes	level at end of treatment	pg	No
6. Gastrointestinal disturbances as side effects	Yes	Vomiting, constipation.	-	No

Section 4. Data and analysis

**DICHOTOMOUS OUTCOME**	Intervention group		Control group	
	Number of events	Number of participants	Number of events	Number of participants
Gastrointestinal disturbances as side effects	7	55	10	64

State details if outcomes were only described in text or figures.

**CONTINUOUS OUTCOME**	Unit of measurement	Intervention group		Control group	
		n	Mean (SD)	n	Mean (SD)
1. Hemoglobin (Hb)	(g/dL)	52	11. 68 (0.11)	53	12.04 (0.09)
2. Serum Ferritin	ng/mL	52	28.7(2.25)	53	32.3 (3.72)
3. Serum iron	ng/dL	52	55.6 (3.09)	53	56.6(3.61)
4. Serum mean corpuscular volume (**MCV**)	fL	52	74.41 (0.53)	53	75.32(0.78)
5. Serum mean corpuscular hemoglobin (**MCH**)	pg	52	24.96 (0.25)	53	26.436 (1.05)

State details if outcomes were only described in text or figures.

Section 5. Other information

	Description as stated in paper
**Key conclusions of study authors**	Ferrous salts and IPC are both suitable medications in this setting. Iron gluconate is more effective than IPC but less tolerable. Physicians should take this into account when deciding on prevention or treatment.
**Results that you calculated using a formula**	-
**References to other relevant studies** *(Did this report include any references to unpublished data from potentially eligible trials not already identified for this review? If yes, give list contact name and details)*	-
**Correspondence required for further study information** *(from whom, what and when)*	-

Sources:

Higgins JPT, Green S (editors). Cochrane Handbook for Systematic Reviews of Interventions Version 5.1.0 [updated March 2011]. The Cochrane Collaboration, 2011.Available from www.cochrane-handbook.org.
