# Supplementary material for: Effectiveness of iron polymaltose complex in treatment and prevention of iron deficiency anemia in children: a systematic review and meta-analysis
Source: PeerJ. 2021 Jan 13;9:e10527. doi: 10.7717/peerj.10527 (PMC7811280; doi:10.7717/peerj.10527)
Supplement: Appendix S7 — A standardized form used to extract important data from the included trial. Randomized control trial 5: 2011 Yasa. [file peerj-09-10527-s011.pdf]

Study Eligibility & Data Collection Form

General Information

**Study ID**<br>*(e.g. author name, year)*	Yasa, 2011
**Form completed by**	Ritzzaleena Rosli Mohd Rosli
**Study author contact details**	ritzz.rosli@student.usm.my
**Publication type**<br>*(e.g. full report, abstract, letter)*	Full report
**List of included publications**	-
**References of similar trial***	-

*This is when the authors published the same study in several reports. All these references to a similar trial should be linked under one *Study ID* in RevMan.

Study eligibility

	Yes	No	Unclear	Further details
**RCT/Quasi/CCT**	/			RCT
**Relevant participants**	/			
**Relevant interventions**	/			
**Relevant outcomes***	/			

*Include only if the presence of outcomes forms the inclusion criterion

If the above answers are ‘YES’, proceed to Section 1.

If any of the above answers are ‘NO*’, record below the information for ‘Excluded studies’

Reason(s) for exclusion
-

Section 1. Characteristics of included studies

This section is to be completed by only one reviewer. State initials: RRMR

**METHODS**	**Descriptions as stated in paper**
**Aim of study** *(e.g. efficacy, equivalence, pragmatic)*	The current study evaluated the efficacy, tolerability, and acceptability of IPC and ferrous sulfate in a cohort of 103 pediatric patients with IDA during a four-month treatment period.
**Design** *(e.g. parallel, crossover, cluster)*	Parallel study comparing Iron Polymaltose and Ferrous Sulfate.<br>Prospective, randomized, open label.
**Unit of allocation** *(by individuals, cluster/ groups or body parts)*	Individuals
**Start & end dates**	During 2009
**Total study duration**	4 months
**Sources of funding** *(including role of funders)*	The paper was prepared by a medical writer, funded by a grant from Vifor Pharma, Switzerland, based on a detailed report provided by the authors.
**Possible conflicts of interest** *(for study authors)*	-

**PARTICIPANTS**	**Description** *(include information for each intervention or comparison group)*
**Population description** *(Company/companies; occupation)*	Pediatric patients
**Setting** *(including location (city, state, country) and single center / multicenter)*	Department of Pediatric Health and Diseases Outpatient Clinics of the University of Istanbul.
**Inclusion criteria**	1. Diagnosis of IDA was based on age-dependent lower limits of normal for Hb and iron status parameters (Table 1)<br>2. Patients with Hb values below normal were tested for transferrin saturation (TSAT), serum iron, and serum ferritin levels. If any of these iron parameters were below normal, the patient was included in the trial
**Exclusion criteria**	-
**Method of recruitment of participants** *(e.g. phone, mail, clinic patients, voluntary)*	Outpatient Clinics

**Total no. randomized**	103
**Clusters** *(if applicable, no., type, no. people per cluster)*	-
**No. randomized per group** *(specify whether no. people or clusters)*	Intervention: 52<br>Control: 51
**No. missing** *(if overall, e.g. exclusions & withdrawals, whether or not missing from analysis)*	Intervention: 0<br>Control: 0
**Reasons missing**	-
**Baseline imbalances**	-
**Age**	7 months to 17 years old
**Sex (proportion)**	42 females 61 males
**Race/Ethnicity**	-
**Other relevant sociodemographic**	-
**Subgroups measured** *(e.g. split by age or sex)*	-
**Subgroups reported**	-

Section 2. Risk of bias assessment

We recommend you refer to and use the method described in the Cochrane Handbook.

This section is completed by two reviewers. State initials: (i) RRMR (ii) NMN

**Domain**	**Risk of bias**<br>Low<br>High<br>Unclear	**Support for judgement**<br>*(include direct quotes where available with explanatory comments)*	**Location in text or source**<br>*(page, table)*
**Random sequence generation**<br>*(selection bias)*	High	*"Randomization was performed by alternating treatment allocation of newly recruited patients on a weekly basis"*	Page2
**Allocation concealment**<br>*(selection bias)*	High	*"patients who were recruited during one week were allocated to one treatment group and those recruited during the following week to the other treatment group."*	Page 2
**Blinding of participants and personnel**<br>*(performance bias)*	unclear	Not mentioned in full text	-
**Blinding of outcome assessment**<br>*(detection bias)*	low	*"Iron parameters were assessed with standard laboratory methods using COBAS INTEGRA 800 and COBAS E autoanalyzer."*<br><br>Comments: results are unlikely to be affected without blinding.	Page 2
**Incomplete outcome data**<br>*(attrition bias)*	low	No missing patients.<br><br>*"One hundred and three children were screened for eligibility, all of whom met the criteria for inclusion and were recruited to the study (42 girls, 61 boys; mean age 6.4 ± 5.1 years, range 7 months to 17 years). The patients were evenly distributed between the two treatment groups (IPC, n = 52, 49.5%; ferrous sulfate, n = 51, 50.5%)."*	Page 2
**Selective outcome reporting**<br>*(reporting bias)*	low	Study protocol not available.<br><br>All pre-specified and expected outcomes of interest are reported	
**Other bias**	low		

Random sequence generation = Process used to assign people into intervention and control groups
Allocation concealment = Process used to prevent foreknowledge of group assignment in a RCT

Blinding of participants and personnel = Presence or absence of blinding for participants and health personnel
Blinding of outcome assessment = presence or absence of blinding for assessment of outcome
Incomplete outcome data = application of intention-to-treat analysis is one in which all the participants in a trial are analyzed according to the intervention to which they were allocated
Selective outcome reporting = Selection of a subset of the original variables recorded

Section 3. Intervention groups

This section is completed by two reviewers. State initials: (i) RRMR (ii) NMN

**Outcomes relevant to your review** *(Copy and paste from 'Types of outcome measures')*	**Reported in paper** *(Yes / No)*	**Outcome definition** *(with diagnostic criteria if relevant)*	**Unit of measurement & tool** *(if relevant)*	**Reanalysis required?** *(specify)*
1. Hemoglobin (Hb)	Yes	Mean level at end of treatment	(g/dL)	No
2. Serum Ferritin	Yes	Mean level at end of treatment	mcg/L	No
3. Serum iron	Yes	Mean level at end of treatment	mcg/dL	No
4. Serum mean corpuscular volume (MCV)	Yes	Mean level at end of treatment	fL	No
5. Serum mean corpuscular hemoglobin (MCH)	Yes	Mean level at end of treatment	pg	No
6. Gastrointestinal disturbances as side effects	Yes	Nausea, abdominal pain, constipation	-	No

Section 4. Data and analysis

**DICHOTOMOUS OUTCOME**	Intervention group		Control group	
	Number of events	Number of participants	Number of events	Number of participants
1. Gastrointestinal disturbances as side effects	14	52	13	51

State details if outcomes were only described in text or figures.

**CONTINUOUS OUTCOME**	Unit of measurement	Intervention group		Control group	
		n	Mean (SD)	n	Mean (SD)
1. Hemoglobin (Hb)	(g/dL)	52	11.7 (0.8)	51	12.4(1)
2. Serum Ferritin	mcg/L	52	33.4 (31)	51	50.3 (67.3)
3. Serum iron	mcg/dL	52	76.3 (60.5)	51	75.7 (36.8)
4. Serum mean corpuscular volume (MCV)	fL	52	76.3 (5.3)	51	79.5 (5.8)
5. Serum mean corpuscular hemoglobin (MCH)	pg	52	25.2 (2.3)	51	26.1 (2.6)

State details if outcomes were only described in text or figures.

Section 5. Other information

	**Description as stated in paper**
**Key conclusions of study authors**	*"The results of this study show that IPC is as effective as ferrous sulfate when used as an oral iron replacement therapy in pediatric patients with iron deficiency anemia. The superior tolerability of IPC compared to ferrous sulfate translated into better treatment acceptability in this population of infants and children."*
**Results that you calculated using a formula**	-
**References to other relevant studies** *(Did this report include any references to unpublished data from potentially eligible trials not already identified for this review? If yes, give list contact name and details)*	-
**Correspondence required for further study information** *(from whom, what and when)*	-

Sources:

Higgins JPT, Green S (editors). Cochrane Handbook for Systematic Reviews of Interventions Version 5.1.0 [updated March 2011]. The Cochrane Collaboration, 2011.Available from www.cochrane-handbook.org.
