# Supplementary material for: Effectiveness of iron polymaltose complex in treatment and prevention of iron deficiency anemia in children: a systematic review and meta-analysis
Source: PeerJ. 2021 Jan 13;9:e10527. doi: 10.7717/peerj.10527 (PMC7811280; doi:10.7717/peerj.10527)
Supplement: Appendix S8 — A standardized form used to extract important data from the included trial. Randomized control trial 6: 2014 Aycicek. [file peerj-09-10527-s012.pdf]

Study Eligibility & Data Collection Form

General Information

**Study ID**<br>*(e.g. author name, year)*	Aycicek, 2014
**Form completed by**	Ritzzaleena Rosli Mohd Rosli
**Study author contact details**	ritzz.rosli@student.usm.my
**Publication type**<br>*(e.g. full report, abstract, letter)*	Full report
**List of included publications**	-
**References of similar trial***	-

*This is when the authors published the same study in several reports. All these references to a similar trial should be linked under one *Study ID* in RevMan.

Study eligibility

	Yes	No	Unclear	Further details
**RCT/Quasi/CCT**	/			RCT
**Relevant participants**	/			
**Relevant interventions**	/			
**Relevant outcomes***	/			

*Include only if the presence of outcomes form the inclusion criterion

If the above answers are 'YES', proceed to Section 1.

If any of the above answers are 'NO*', record below the information for 'Excluded studies'

Reason(s) for exclusion
-

Section 1. Characteristics of included studies

This section is to be completed by only one reviewer. State initials: RRMR

METHODS	Descriptions as stated in paper
**Aim of study** *(e.g. efficacy, equivalence, pragmatic)*	The purpose of this study was to compare the total oxidant and antioxidant effect of different oral iron preparations in children with iron-deficiency anemia (IDA).
**Design** *(e.g. parallel, crossover, cluster)*	Parallel study comparing Iron Polymaltose, Ferrous Sulphate and healthy children without any treatment.<br>Open label randomized controlled trial.
**Unit of allocation** *(by individuals, cluster/ groups or body parts)*	individuals
**Start & end dates**	January 2011 until May 2012
**Total study duration**	17 months
**Sources of funding** *(including role of funders)*	-
**Possible conflicts of interest** *(for study authors)*	-

PARTICIPANTS	**Description**<br>*(include information for each intervention or comparison group)*
**Population description** *(Company/companies; occupation)*	Pediatrics patients
**Setting** *(including location (city, state, country) and single center / multicenter)*	Pediatrics and Pediatric Hematology Outpatient Clinic in Harran University, Turkey.
**Inclusion criteria**	1. IDA was defined as hemoglobin (Hb) below 10.6 g/dL for children at or below the age of 2 years and below 11 g/dL for children older than 2 years and with a serum ferritin value below 12 ng/mL.<br>2. Aged between 1 and 16 years.
**Exclusion criteria**	1. if they had used iron preparations in the previous 3 months<br>2. Had acute infection<br>3. Had a history of chronic disease or parasites<br>4. Suffered blood loss for any reason<br>5. Had occult blood in their stools
**Method of recruitment of participants** *(e.g. phone, mail, clinic patients, voluntary)*	All patients were seen at the Outpatient Service of Pediatric Hematology Department

**Total no. randomized**	72
**Clusters** *(if applicable, no., type, no. people per cluster)*	-
**No. randomized per group** *(specify whether no. people or clusters)*	Intervention (IPC): 33<br>Comparison (Ferrous sulphate): 32<br>Control (healthy, no treatment): 28
**No. missing** *(if overall, e.g. exclusions & withdrawals, whether or not missing from analysis)*	7 from overall (loss to follow up)<br>10 excluded after assessment of proper drug used (Intervention:5. Comparison:5)
**Reasons missing**	Loss to follow up, improper use of drugs
**Baseline imbalances**	No
**Age**	1-16 years old
**Sex (proportion)**	-
**Race/Ethnicity**	-
**Other relevant sociodemographic**	-
**Subgroups measured** *(e.g. split by age or sex)*	-
**Subgroups reported**	-

Section 2. Risk of bias assessment

We recommend you refer to and use the method described in the Cochrane Handbook.

This section is completed by two reviewers. State initials: (i) RRMR (ii) NMN

**Domain**	**Risk of bias**<br><br>Low High Unclear	**Support for judgement**<br>*(include direct quotes where available with explanatory comments)*	**Location in text or source**<br>*(page, table)*
**Random sequence generation**<br>*(selection bias)*	low	*"simple randomization with no restrictions or matching"*	Page 1
**Allocation concealment**<br>*(selection bias)*	unclear	Not mentioned in full text	-
**Blinding of participants and personnel**<br>*(performance bias)*	unclear	Not mentioned in full text	-
**Blinding of outcome assessment**<br>*(detection bias)*	low	*"All analyses were performed at a single laboratory"*<br><br>Comments: results are unlikely to be affected without blinding.	Page 2
**Incomplete outcome data**<br>*(attrition bias)*	low	Number of missing participants are equal in each group and both for similar reason.	Page 2
**Selective outcome reporting**<br>*(reporting bias)*	low	Study protocol not available.<br><br>All pre-specified and expected outcomes of interest are reported	
**Other bias**	low	No other bias identified	

Random sequence generation = Process used to assign people into intervention and control groups
Allocation concealment = Process used to prevent foreknowledge of group assignment in a RCT
Blinding of participants and personnel = Presence or absence of blinding for participants and health personnel
Blinding of outcome assessment = presence or absence of blinding for assessment of outcome
Incomplete outcome data = application of intention-to-treat analysis is one in which all the participants in a trial are analyzed according to the intervention to which they were allocated
Selective outcome reporting = Selection of a subset of the original variables recorded

Section 3. Intervention groups

This section is completed by two reviewers. State initials: (i) RRMR (ii) NMN

**Outcomes relevant to your review** *(Copy and paste from 'Types of outcome measures')*	**Reported in paper** *(Yes / No)*	**Outcome definition** *(with diagnostic criteria if relevant)*	**Unit of measurement & tool** *(if relevant)*	**Reanalysis required?** *(specify)*
1. Hemoglobin (Hb)	Yes	Mean level at end of treatment	(g/dL) automated analyser (Celldyn 3700; Abbott, IL).	No
2. Serum Ferritin	Yes	Mean level at end of treatment	ng/mL<br>commercial kits (Abbott)	No
3. Serum iron	Yes	Mean level at end of treatment	mcg/dL<br>commercial kits (Abbott)	No
4. Serum mean corpuscular volume (MCV)	No	-	-	No
5. Serum mean corpuscular hemoglobin (MCH)	No	-	-	No
6. Gastrointestinal disturbances as side effects	Yes	Nausea, abdominal pain.	-	No

Section 4. Data and analysis

DICHOTOMOUS OUTCOME	Intervention group		Control group	
	Number of events	Number of participants	Number of events	Number of participants
1. Gastrointestinal disturbances as side effects	4	32	7	33

State details if outcomes were only described in text or figures.

CONTINUOUS OUTCOME	Unit of measurement	Intervention group		Control group	
		n	Mean (SD)	n	Mean (SD)
1. Hemoglobin (Hb)	(g/dL)	27	9.6 (1.8)	28	11.4 (1.4)
2. Serum Ferritin	ng/mL	27	18.7 (20.1)	28	29.4 (22.4)
3. Serum iron	mcg/dL	27	28.4 (14.8)	28	45.9 (22.4)

State details if outcomes were only described in text or figures.

Section 5. Other information

	**Description as stated in paper**
**Key conclusions of study authors**	*"Serum total oxidant and antioxidant was significantly increased in children with IDA, and ferrous sulphate was highly effective in correcting elevated oxidative status."*
**Results that you calculated using a formula**	-
**References to other relevant studies** *(Did this report include any references to unpublished data from potentially eligible trials not already identified for this review? If yes, give list contact name and details)*	-
**Correspondence required for further study information** *(from whom, what and when)*	-

Sources:
Higgins JPT, Green S (editors). Cochrane Handbook for Systematic Reviews of Interventions Version 5.1.0 [updated March 2011]. The Cochrane Collaboration, 2011.Available from www.cochrane-handbook.org.
