# Supplementary material for: Effectiveness of iron polymaltose complex in treatment and prevention of iron deficiency anemia in children: a systematic review and meta-analysis
Source: PeerJ. 2021 Jan 13;9:e10527. doi: 10.7717/peerj.10527 (PMC7811280; doi:10.7717/peerj.10527)
Supplement: Appendix S9 — A standardized form used to extract important data from the included trial. Randomized control trial 7: 2015 Ozsurekci. [file peerj-09-10527-s013.pdf]

Study Eligibility & Data Collection Form

General Information

**Study ID**<br>*(e.g. author name, year)*	Ozsurekci, 2015
**Form completed by**	Ritzzaleena Rosli Mohd Rosli
**Study author contact details**	ritzz.rosli@student.usm.my
**Publication type**<br>*(e.g. full report, abstract, letter)*	Full report
**List of included publications**	-
**References of similar trial***	-

*This is when the authors published the same study in several reports. All these references to a similar trial should be linked under one *Study ID* in RevMan.

Study eligibility

	Yes	No	Unclear	Further details
**RCT/Quasi/CCT**	/			RCT
**Relevant participants**	/			
**Relevant interventions**	/			
**Relevant outcomes***	/			

*Include only if the presence of outcomes forms the inclusion criterion

If the above answers are 'YES', proceed to Section 1.

If any of the above answers are 'NO*', record below the information for 'Excluded studies'

Reason(s) for exclusion
-

Section 1. Characteristics of included studies

This section is to be completed by only one reviewer. State initials: RRMR

METHODS	Descriptions as stated in paper
**Aim of study** *(e.g. efficacy, equivalence, pragmatic)*	The purpose of this study was to compare the effectiveness of different oral iron preparations in children with iron deficiency anemia (IDA)
**Design** *(e.g. parallel, crossover, cluster)*	Parallel study of 3 treatment groups. Group I received ferrous sulfate (Fe-S); Group II received iron polymaltose complexes (Fe-OH-PM), and Group III received a single preparation of combined iron and zinc (Fe-Zn).
**Unit of allocation** *(by individuals, cluster/ groups or body parts)*	Individuals
**Start & end dates**	January 2008 till March 2009
**Total study duration**	1 year 3 months
**Sources of funding** *(including role of funders)*	-
**Possible conflicts of interest** *(for study authors)*	-

PARTICIPANTS	**Description**<br>*(include information for each intervention or comparison group)*
**Population description** *(Company/companies; occupation)*	Children with iron deficiency anemia
**Setting** *(including location (city, state, country) and single center / multicenter)*	Pediatric outpatient clinic in Hecettepe University Faculty of Medicine, Pediatric Hematology Unit.
**Inclusion criteria**	1. Anemia was defined as hemoglobin (Hgb) below -2SD according to age and gender.<br>2. Iron deficiency (ID) was defined with serum iron, iron-binding capacity, ferritin, and transferrin saturation levels below the range for age and gender appropriates.
**Exclusion criteria**	1. Anemia due to other causes except IDA; Severe concurrent illness (cardiovascular, renal, hepatic)<br>2. Known hypersensitivity to ferrous or ferric preparations<br>3. Malignancy of any types<br>4. Children with thalassemia major<br>5. Sickle cell anemia or other haemoglobinopathies<br>6. Hemolytic anemia or aplastic or hypoplastic anemia.

**Method of recruitment of participants** *(e.g. phone, mail, clinic patients, voluntary)*	consecutive patients who attended the pediatric outpatient clinic.
**Total no. randomized**	60
**Clusters** *(if applicable, no., type, no. people per cluster)*	No
**No. randomized per group** *(specify whether no. people or clusters)*	Intervention: 21<br>Control: 19<br>(group III: 20)
**No. missing** *(if overall, e.g. exclusions & withdrawals, whether or not missing from analysis)*	15 dropped out during course of the study.
**Reasons missing**	Discontinuation of follow up
**Baseline imbalances**	No
**Age**	6 months to 15 years
**Sex (proportion)**	35 boys 25 girls
**Race/Ethnicity**	-
**Other relevant sociodemographic**	-
**Subgroups measured** *(e.g. split by age or sex)*	-
**Subgroups reported**	-

Section 2. Risk of bias assessment

We recommend you refer to and use the method described in the Cochrane Handbook.

This section is completed by two reviewers. State initials: (i)RRMR (ii) NMN

**Domain**	**Risk of bias**<br>Low<br>High<br>Unclear	**Support for judgement**<br>*(include direct quotes where available with explanatory comments)*	**Location in text or source** *(page, table)*
**Random sequence generation** *(selection bias)*	high	*were randomly included in Fe-S (group I), Fe-OH (group II), and Ferro Zinc(group III) in a consecutive fashion.*	Page2
**Allocation concealment** *(selection bias)*	unclear	*Not mentioned in full text*	-
**Blinding of participants and personnel** *(performance bias)*	unclear	Not mentioned in full text	-
**Blinding of outcome assessment** *(detection bias)*	low	Comment: Result are unlikely to be affected.<br><br>*"Complete blood count (CBC), peripheral blood smear and reticulocyte levels were ordered on the same day"*	-
**Incomplete outcome data** *(attrition bias)*	low	No missing patients after randomization done.	-
**Selective outcome reporting** *(reporting bias)*	low	Study protocol not available. However, all pre-specified and expected outcomes of interest are reported	-
**Other bias**	low	No other bias detected	-

Random sequence generation = Process used to assign people into intervention and control groups
Allocation concealment = Process used to prevent foreknowledge of group assignment in a RCT
Blinding of participants and personnel = Presence or absence of blinding for participants and health personnel
Blinding of outcome assessment = presence or absence of blinding for assessment of outcome
Incomplete outcome data = application of intention-to-treat analysis is one in which all the participants in a trial are analyzed according to the intervention to which they were allocated
Selective outcome reporting = Selection of a subset of the original variables recorded

Section 3. Intervention groups

This section is completed by two reviewers. State initials: (i) RRMR (ii) NMN

**Outcomes relevant to your review** *(Copy and paste from 'Types of outcome measures')*	**Reported in paper** *(Yes / No)*	**Outcome definition** *(with diagnostic criteria if relevant)*	**Unit of measurement & tool** *(if relevant)*	**Reanalysis required?** *(specify)*
1. Hemoglobin (Hb)	Yes	Mean level at end of treatment	(g/dL)	No
2. Serum Ferritin	No	-	-	No
3. Serum iron	No	-	-	No
4. Serum mean corpuscular volume (MCV)	No	-	-	No
5. Serum mean corpuscular hemoglobin (MCH)	No	-	-	No
6. Gastrointestinal disturbances as side effects	No	-	-	No

Section 4. Data and analysis

**DICHOTOMOUS OUTCOME**	Intervention group		Control group	
	Number of events	Number of participants	Number of events	Number of participants
1.	-	-	-	-

State details if outcomes were only described in text or figures.

**CONTINUOUS OUTCOME**	Unit of measurement	Intervention group		Control group	
		n	Mean (SD)	n	Mean (SD)
1. Hemoglobin (Hb)	(g/dL)	19	11.2 (0.5)	21	11.6(0.8)

State details if outcomes were only described in text or figures.

Section 5. Other information

	**Description as stated in paper**
**Key conclusions of study authors**	*"each of the three iron preparations have been found to be effective in correcting IDA. There is no difference between the groups"*<br>*"in all three groups, no severe side effects were observed"*<br>*"both Fe-OH-PM and Fe-Zn preparations like Fe-S may be a choice in the treatment of children with IDA."*
**Results that you calculated using a formula**	-
**References to other relevant studies** *(Did this report include any references to unpublished data from potentially eligible trials not already identified for this review? If yes, give list contact name and details)*	-
**Correspondence required for further study information** *(from whom, what and when)*	-

Sources:

Higgins JPT, Green S (editors). Cochrane Handbook for Systematic Reviews of Interventions Version 5.1.0 [updated March 2011]. The Cochrane Collaboration, 2011.Available from www.cochrane-handbook.org.
