# Supplementary material for: Effectiveness of iron polymaltose complex in treatment and prevention of iron deficiency anemia in children: a systematic review and meta-analysis
Source: PeerJ. 2021 Jan 13;9:e10527. doi: 10.7717/peerj.10527 (PMC7811280; doi:10.7717/peerj.10527)
Supplement: Appendix S10 — A standardized form used to extract important data from the included trial. Randomized control trial 8: 2018 Name. [file peerj-09-10527-s014.pdf]

Study Eligibility & Data Collection Form

General Information

**Study ID**<br>*(e.g. author name, year)*	Name, 2018
**Form completed by**	Ritzzaleena Rosli Mohd Rosli
**Study author contact details**	ritzz.rosli@student.usm.my
**Publication type**<br>*(e.g. full report, abstract, letter)*	Full report
**List of included publications**	-
**References of similar trial***	-

*This is when the authors published the same study in several reports. All these references to a similar trial should be linked under one *Study ID* in RevMan.

Study eligibility

	Yes	No	Unclear	Further details
**RCT/Quasi/CCT**	/			RCT
**Relevant participants**	/			
**Relevant interventions**	/			
**Relevant outcomes***	/			

*Include only if the presence of outcomes forms the inclusion criterion

If the above answers are 'YES', proceed to Section 1.

If any of the above answers are 'NO*', record below the information for 'Excluded studies'

Reason(s) for exclusion
-

Section 1. Characteristics of included studies

This section is to be completed by only one reviewer. State initials: RRMR

METHODS	Descriptions as stated in paper
**Aim of study** *(e.g. efficacy, equivalence, pragmatic)*	To compare the efficacy of oral supplementation with iron bisglycinate Chelate and Iron polymaltose in anaemic children
**Design** *(e.g. parallel, crossover, cluster)*	Parallel study comparing iron bisglycinate chelate and iron polymaltose
**Unit of allocation** *(by individuals, cluster/ groups or body parts)*	Individuals
**Start & end dates**	July 2016 to December 2016
**Total study duration**	6 months
**Sources of funding** *(including role of funders)*	Not specified. However, the Sensitiva Compounding Pharmacy has kindly prepared the iron supplements.
**Possible conflicts of interest** *(for study authors)*	-

PARTICIPANTS	**Description** *(include information for each intervention or comparison group)*
**Population description** *(Company/companies; occupation)*	Anemic children.
**Setting** *(including location (city, state, country) and single center / multicenter)*	Reino da Garotada, a non-profit institution in the city of Poá, São Paulo state, Brazil.
**Inclusion criteria**	1. Children who had confirmed haemoglobin levels below the WHO criterion by means of complete blood count, ferritin and transferrin.
**Exclusion criteria**	1. Those treated with drugs that interfered with iron absorption 60 days before the start of treatment.<br>2. Those supplemented with iron 60 days before the start of treatment.<br>3. The participants had any signs of infection (fever, vomiting or diarrhoea) on blood collection days.<br>4. Children weighing over 30 kg were also excluded
**Method of recruitment of participants** *(e.g. phone, mail, clinic patients, voluntary)*	Not mentioned
**Total no. randomized**	20

**Clusters** *(if applicable, no., type, no. people per cluster)*	No
**No. randomized per group** *(specify whether no. people or clusters)*	Intervention: people =11<br>Control: people = 9
**No. missing** *(if overall, e.g. exclusions & withdrawals, whether or not missing from analysis)*	Intervention: 0<br>Control: 0
**Reasons missing**	Intervention: -<br>Control: -
**Baseline imbalances**	No
**Age**	1-13 years old
**Sex (proportion)**	Intervention: Male 7 female 4<br>Control: Male 6 Female 3
**Race/Ethnicity**	Not mentioned
**Other relevant sociodemographic**	Precarious social and economic conditions
**Subgroups measured** *(e.g. split by age or sex)*	-
**Subgroups reported**	-

Section 2. Risk of bias assessment

We recommend you refer to and use the method described in the Cochrane Handbook.

This section is completed by two reviewers. State initials: (i) RRMR (ii) NMN

**Domain**	**Risk of bias**<br>Low<br>High<br>Unclear	**Support for judgement**<br>*(include direct quotes where available with explanatory comments)*	**Location in text or source**<br>*(page, table)*
**Random sequence generation**<br>*(selection bias)*	low	*"Children diagnosed with IDA were randomized using computer generated random numbers,"*	Page 3
**Allocation concealment**<br>*(selection bias)*	low	*"by an independent statistician, who was not an investigator, into 2 groups"*	Page 3
**Blinding of participants and personnel**<br>*(performance bias)*	low	*"A trained nurse technician administered the treatments to each child after the main meal as a single daily dose for 45 days. Participating children, care providers and investigators who accessed outcomes were blind to the exact intervention administered. Unblinding was performed only after completion of the study"*	Page 3
**Blinding of outcome assessment**<br>*(detection bias)*	low	*"Portable haemoglobin analyzer, HemoCue Hb301 (HemoCue AB, Ängelholm, Sweden), was used to measure hemoglobin levels. Blood samples were collected by digital puncture. Transferrin levels were measured by immunoturbidimetry, and ferritin was measured by chemiluminescence (Siemens Healthcare Diagnostics, Deerfield, IL, USA). MCV, MCH and RDW were measured by standard clinical laboratory methods (XE 2000, Roche Diagnostics, Mann heim, Germany)"*	Page 3
**Incomplete outcome data**<br>*(attrition bias)*	low	No missing patients.<br><br>*"Thus, a total of 20 children, aged 1-13 years, participated in the study"*	Page 3
**Selective outcome reporting**<br>*(reporting bias)*	low	Unable to retrieved full text of trial. Universal Trial Number U1111-1216-2727<br><br>All pre-specified and expected outcomes of interest are reported	-
**Other bias**	low	No other bias detected.	-

Random sequence generation = Process used to assign people into intervention and control groups
Allocation concealment = Process used to prevent foreknowledge of group assignment in a RCT
Blinding of participants and personnel = Presence or absence of blinding for participants and health personnel
Blinding of outcome assessment = presence or absence of blinding for assessment of outcome
Incomplete outcome data = application of intention-to-treat analysis is one in which all the participants in a trial are analyzed according to the intervention to which they were allocated
Selective outcome reporting = Selection of a subset of the original variables recorded

Section 3. Intervention groups

This section is completed by two reviewers. State initials: (i) RRMR (ii) NMN

**Outcomes relevant to your review** *(Copy and paste from 'Types of outcome measures')*	**Reported in paper** *(Yes / No)*	**Outcome definition** *(with diagnostic criteria if relevant)*	**Unit of measurement & tool** *(if relevant)*	**Reanalysis required?** *(specify)*
1. Hemoglobin (Hb)	Yes	Mean level at end of treatment	(g/dL)	No
2. Serum Ferritin	Yes	Mean level at end of treatment	ng/mL	No
3. Serum iron	No	-	mcg/dL	No
4. Serum mean corpuscular volume (MCV)	Yes	Mean level at end of treatment	fL	No
5. Serum mean corpuscular hemoglobin (MCH)	Yes	Mean level at end of treatment	pg	No
6. Gastrointestinal disturbances as side effects	No	-	-	No

Section 4. Data and analysis

DICHOTOMOUS OUTCOME	Intervention group		Control group	
	Number of events	Number of participants	Number of events	Number of participants
1. -	-	-	-	-

State details if outcomes were only described in text or figures.

CONTINUOUS OUTCOME	Unit of measurement	Intervention group		Control group	
		n	Mean (SD)	n	Mean (SD)
1. Hemoglobin (Hb)	(g/dL)	11	12.2 (0.2)	9	12.2 (0.30)
2. Serum Ferritin	ng/mL	11	34 (3.9)	9	37 (5)
3. Serum mean corpuscular volume (MCV)	fL	11	76.1(1.3)	9	74.2 (2.3)
4. Serum mean corpuscular hemoglobin (MCH)	pg	11	25.5(0.5)	9	24.4(1)

State details if outcomes were only described in text or figures.

Section 5. Other information

	**Description as stated in paper**
**Key conclusions of study authors**	*"The present work provides preliminary evidence to suggest that iron bisglycinate chelate (standard treatment) is more effective than iron polymaltose complex (intervention) in increasing iron stores in the body. Moreover, the results suggest that, in contrast to iron polymaltose complex, iron bisglycinate chelate absorption is proportional to iron demand, showing it to be a safe compound for treating IDA."*
**Results that you calculated using a formula**	-
**References to other relevant studies** *(Did this report include any references to unpublished data from potentially eligible trials not already identified for this review? If yes, give list contact name and details)*	-
**Correspondence required for further study information** *(from whom, what and when)*	-

Sources:
Higgins JPT, Green S (editors). Cochrane Handbook for Systematic Reviews of Interventions Version 5.1.0 [updated March 2011]. The Cochrane Collaboration, 2011.Available from www.cochrane-handbook.org.
