# Supplementary material for: Effectiveness of iron polymaltose complex in treatment and prevention of iron deficiency anemia in children: a systematic review and meta-analysis
Source: PeerJ. 2021 Jan 13;9:e10527. doi: 10.7717/peerj.10527 (PMC7811280; doi:10.7717/peerj.10527)
Supplement: File S1 [file peerj-09-10527-s015.docx]

**The rationale and significance for conducting this systematic review and meta-analysis.**

Two previously published meta-analyses investigated the effectiveness and tolerability of ferrous sulphate (FS) in patients with IDA (Toblli & Brignoli 2007; Tolkien et al. 2015). However, one review compared the tolerability of FS to a placebo group (Tolkien et al. 2015). The review explored the gastrointestinal side effects in adult patients and confirmed that FS significantly increased the occurrence of gastrointestinal side effects compared to the placebo group at any dose.

Another systematic review studied the efficacy of IPC compared to FS in the treatment of IDA for all populations (Toblli & Brignoli 2007). However, this review excluded pediatric trials from the meta-analysis because the pediatric groups were not comparable at baseline. The same review reported that the dropout rates were similar in the IPC and FS groups in the trials for both adults and children. However, the adverse drug reactions were significantly less frequent among the adults and children treated with IPC.

A meta-analysis in 2015 showed that FS increased gastrointestinal disturbances with an odds ratio of 2.32 (Tolkien et al. 2015). The most frequently reported gastrointestinal disturbances include nausea, vomiting, colicky abdominal pain, gastritis, tarry stool, and diarrhea (Cancelo-Hidalgo et al. 2013; DeLoughery 2019; Tolkien et al. 2015).

Therefore, the objectives of this study were to determine the efficacy and safety of oral iron polymaltose complex (IPC) for the prevention and treatment of iron deficiency anemia (IDA) in infants, children, and adolescents. The aim was to provide high-quality evidence comparing oral IPC and oral ferrous iron commonly used in routine practice.

REFERENCES:

Toblli JE, and Brignoli R. 2007. Iron (III)-hydroxide polymaltose complex in iron deficiency anemia / review and meta-analysis. *Drug Res* 57:431-438. 10.1055/s-0031-1296692

Tolkien Z, Stecher L, Mander AP, Pereira DI, and Powell JJ. 2015. Ferrous sulfate supplementation causes significant gastrointestinal side-effects in adults: a systematic review and meta-analysis. *PLoS One* 10:e0117383. 10.1371/journal.pone.0117383
